# Neural and Cardiac Contributions to Perceptual Suppression During Cycling

**DOI:** 10.1111/psyp.70144

**Published:** 2025-09-09

**Authors:** Aishwarya Bhonsle, Melanie Wilke

**Affiliations:** ^1^ Department of Cognitive Neurology University Medical Center Göttingen Göttingen Germany; ^2^ MR‐Research in Neurosciences University Medical Center Göttingen Göttingen Germany; ^3^ Cognitive Neurology Group, Department of Cognitive Neuroscience German Primate Center, Leibniz Institute for Primate Research Göttingen Germany

## Abstract

Exercise influences visual processing and is accompanied by neural and physiological changes in the body. Yet, the underlying mechanisms by which neural and physiological responses to exercise impact ensuing perception remain poorly understood. In particular, the effects of exercise‐induced cardiac changes on visual perception and electrophysiological activity are unclear. Here, we aimed to investigate the relationship between conscious visual perception, neural activity, and cardiac responses during exercise. Thirty healthy participants performed a perceptual suppression task while engaging in light‐intensity stationary cycling, with EEG and ECG activity recorded simultaneously. Our study shows that the probability of perceptual suppression decreased during cycling. Parieto‐occipital alpha amplitudes (8–12 Hz) also decreased during cycling, but this reduction did not correlate with the decrease in perceptual suppression. Additionally, cycling decreased heartbeat‐evoked potential (HEP) amplitudes, indicating altered neural processing of cardiac signals during exercise. However, these exercise‐induced changes in HEP amplitudes did not predict perceptual outcomes. Moreover, changes in heart rate in response to cycling did not correlate with changes in perceptual suppression rates, pre‐random dot motion stimulus alpha, or HEP amplitudes. These findings indicate that while exercise modulates conscious visual perception, the associated changes in alpha activity, heart rate, and HEPs do not fully explain this effect. Our results highlight the complex relationship between interoceptive processing and mechanisms underlying the perception of external stimuli during exercise.

## Impact Statement

Our study investigated how light‐intensity cycling impacts perceptual suppression and pre‐stimulus parieto‐occipital alpha activity and heartbeat‐evoked potentials (HEPs). Exercise decreased perceptual suppression rates, occipital alpha, and HEP amplitudes. We tested the theory that exercise influences neural activity through baroreceptor‐driven cortical excitability, and our findings challenge this mechanism. Additionally, we provide novel evidence of exercise‐induced changes in HEPs, reflecting shifts in neural processing of cardiac signals and revealing a potential influence of cardiac physiology on HEPs.

## 1 Introduction

A single bout of exercise can produce positive effects on cognitive function (Lambourne and Tomporowski 2010; Chang et al. 2012; Cantelon and Giles 2021; Garrett et al. 2024), alongside various neural (Crabbe and Dishman 2004; Hosang et al. 2022) and bodily physiological responses (Heinonen et al. 2014). Studies of human visual processing during physical activity reveal that exercise‐induced neural changes are linked to altered perceptual dynamics, which differ markedly from those observed at rest (Cao and Händel 2019; Bullock et al. 2017, 2015; De Sanctis et al. 2014). Among these neural changes, cortical oscillatory activity across the entire frequency spectrum and various brain regions shows consistent modulation during acute bouts of physical activity, with findings in the alpha band (8–12 Hz) being the most consistent, though the direction and location of these effects vary (see refs. (Crabbe and Dishman 2004; Hosang et al. 2022) for reviews). These neural responses are highly context‐dependent, as variations in the combinations of perceptual tasks and types of physical activity contribute to differences in the observed outcomes. For example, naturalistic walking has been linked to a decrease in alpha power, which is thought to reflect reduced inhibitory processes that typically suppress peripheral visual input (Cao and Händel 2019). During an oddball task, moderate‐to‐high intensity cycling led to a smaller decrease in parieto‐occipital alpha power in response to non‐target stimuli compared to light exercise, while detection accuracy remained consistent across intensities (Ciria et al. 2019). In contrast, increased parieto‐occipital alpha has also been observed during cycling, coinciding with reduced accuracy in an orientation discrimination task (Bullock et al. 2017). Independent of perceptual task demands, broadband decreases in alpha activity have been observed during cycling and walking, with more pronounced reductions during walking (Storzer et al. 2016). Conversely, increases in alpha activity during cycling have also been observed throughout the brain (Hottenrott et al. 2013), though most frequently across anterior regions (Hosang et al. 2022; Enders et al. 2016; Bailey et al. 2008; Fumoto et al. 2010).

In addition to these neural and perceptual responses, exercise elicits a gamut of physiological responses across virtually all tissues and organs in the human body, including an increase in cardiac activity (Heinonen et al. 2014), an increase in heart rate and a decrease in heart rate variability (HRV) (Mongin et al. 2022). In addition, mild exercise has been shown to increase cerebral blood flow (Querido and Sheel 2007) and neurotransmitter levels of dopamine, norepinephrine, and serotonin (Meeusen and De Meirleir 1995), up to boosting neurotrophic factor (e.g., BDNF) levels (Ferris et al. 2007). While the effects of exercise on oscillatory neural activity and visual perception are the subject of ongoing investigation, the influence of exercise‐induced cardiac changes on neural activity and subsequent perception remains largely understudied. Typically, studies investigating the effects of physical activity have used heart rate as a metric of exercise intensity (Garber et al. 2011), while EEG analyses have considered heart activity as a source of non‐cerebral artifacts, especially under physical exertion.

Heartbeats are transduced into neural signals by mechanoreceptors, which are located within the atria and ventricles of the heart as well as in various blood vessels (Bishop et al. 1983; Jänig 1996; Zeng et al. 2018). Of these, baroreceptors are a type of stretch receptor located in the heart and blood vessels. They are particularly well‐studied for their role in the baroreflex, which regulates heart rate and blood pressure (Berntson et al. 2007; Dampney et al. 2003). Baroreceptors relay information to the brain about the timing and strength of cardiac contractions and transient changes in blood pressure (Bishop et al. 1983). An increase in heart rate leads to increased baroreceptor activity, although there are important exceptions to this rule, for example, in the context of respiratory sinus arrhythmia (RSA) (Noble and Hochman 2019; Berntson et al. 1993).

In addition to baroreceptor inputs, cardiac activity is also signaled to the CNS through alternative pathways. For instance, vessel pulsatility and changes in thoracic volume are detected by tactile and proprioceptive receptors (Macefield 2003; Birznieks et al. 2012; Ford and Kirkwood 2018). Recent findings in rodent models indicate that astrocytes in the CNS may act as intracranial baroreceptors, influencing both brainstem activity and cortical excitability (Kim et al. 2016; Marina et al. 2020). In addition, certain neuron types, such as pyramidal neurons (Kim et al. 2016; Wang and Hamill 2021), and mitral cells in the olfactory bulb (Jammal Salameh et al. 2024), express PIEZO2 channels, which are mechanosensitive ion channels that transduce mechanical stimuli, such as membrane tension, into electrical and biochemical signals (Kefauver et al. 2020). Thus, they may directly detect intracranial pressure pulses, offering yet another route for cardiac signal transduction.

It has been theorized (Crabbe and Dishman 2004; Hosang et al. 2022) that exercise‐induced increases in alpha activity might arise from cortical inhibition driven by brainstem and subcortical activation linked to cardiovascular regulation (Koriath et al. 1987; Lacey and Lacey 1978). This proposal brings together two key concepts: the baroreceptor hypothesis (Lacey and Lacey 1978, 1974, 1958, 1970; Lacey 1967) and alpha activity as an index of cortical excitability (Klimesch et al. 2007; Romei, Rihs, et al. 2008; Romei, Brodbeck, et al. 2008; Jensen and Mazaheri 2010; Iemi et al. 2017). The baroreceptor hypothesis suggests that changes in cardiovascular activity influence cortical excitability via inhibitory afferent feedback from baroreceptors. According to this hypothesis, higher heart rates and increases in baroreceptor firing, should lead to an increase in inhibitory feedback and a subsequent reduction in cortical excitability. In parallel, alpha activity is regarded as an index of cortical excitability, with higher alpha levels reflecting reduced cortical excitability (Klimesch et al. 2007; Romei, Rihs, et al. 2008; Romei, Brodbeck, et al. 2008; Jensen and Mazaheri 2010; Iemi et al. 2017) and visual attentiveness (Kelly et al. 2006; Thut et al. 2006; Sauseng et al. 2005). Alpha‐driven cortical inhibition as a mechanism of neural modulation around the periodic baroreceptor signal has yet to be directly investigated, especially in the context of exercise‐induced cardiovascular changes.

Changes in heart rate are also thought to be a key physiological response mediating the effect of exercise on perceptual and cognitive performance (Chang et al. 2012; Cantelon and Giles 2021). Although this relationship is not well characterized in the context of exercise, existing evidence highlights a link between heart rate dynamics and visual perception. For instance, individuals with lower heart rates demonstrate increased visual stimulus detection accuracy (Sandman et al. 1977). Additionally, studies employing perceptual paradigms performed at rest reveal that heart rate changes occur both prior to and following perception of an external stimulus (Lacey and Lacey 1978, 1974, 1980; Lacey 1967; Jennings and Wood 1977; Somsen et al. 2004; Park et al. 2014; Cobos et al. 2019; Motyka et al. 2019; Grund et al. 2022; Vila et al. 2007; Skora et al. 2022). Specifically, heart rate typically decelerates in anticipation of an upcoming stimulus, a pattern thought to reflect sustained attention and preparatory processes (Lacey and Lacey 1978; Vila et al. 2007; Skora et al. 2022), and then accelerates again, following stimulus detection or response registration.

These instantaneous adjustments in heart rate are thought to fine‐tune the balance between internal bodily signals (interoception) and external sensory inputs (exteroception), thereby facilitating perception and action (Skora et al. 2022). Cardiac deceleration has been proposed to reduce the inhibitory influence of baroreceptor activity, enhance attention to external stimuli, and improve sensory processing and perception. Consistent with this notion, preliminary evidence shows that cardiac deceleration tracks active attention during binocular rivalry (Corcoran et al. 2021). Conversely, heart rate acceleration following response registration restores the balance between interoceptive and exteroceptive processing and prepares the system for action (Skora et al. 2022).

Interestingly, this competition between interoception and exteroception, independent of cardiovascular mechanisms, has also been discussed in the context of exercise, where shifts in attentional focus between these domains are thought to modulate the perception of fatigue (Pennebaker and Lightner 1980; Wallman‐Jones et al. 2021). However, while resting heart rates and instantaneous heart rate changes have been linked to the perception of visual stimuli and are thought to reflect an interoceptive‐exteroceptive trade‐off, how sustained heart rate increases during exercise influence visual perceptual dynamics, particularly in relation to alpha oscillations, remains unexplored.

Moving beyond heart rate and alpha activity changes, heartbeat‐evoked potentials (HEP) are a neural measure that has not yet been investigated in the context of exercise. HEPs represent transient neural activity observed when electrophysiological data are time‐locked to heartbeats and are thought to serve as a marker of cortical processing of cardiac information (Schandry and Montoya 1996; Gray et al. 2007; Park and Blanke 2019; Coll et al. 2021). HEP amplitudes are increased during interoceptive tasks such as heartbeat counting (Montoya et al. 1993; Petzschner et al. 2019). HEP amplitudes have also been shown to predict conscious perception of visual (Park et al. 2014) and somatosensory (Al et al. 2020) stimuli at threshold, further linking interoceptive and exteroceptive processing. Investigating the effect of cycling on HEPs could offer valuable insights into how exercise modulates heart‐brain coupling, potentially illuminating interactions between cardiac and cortical responses. Furthermore, examining whether cycling‐related modulation of HEPs influences conscious perception could provide additional insight into the contribution of interoceptive processing to exteroceptive perceptual awareness.

In this study, we investigated the neural and cardiac changes through which exercise influences conscious visual perception in a bistable perceptual suppression paradigm. Specifically, we examined how stationary cycling modulates oscillatory brain activity, heart rate, and heart‐brain coupling, and how these responses potentially influence conscious perception in a Generalized Flash Suppression (GFS) paradigm. GFS is a perceptual suppression paradigm in which a salient target is rendered subjectively invisible upon presentation of a random dot motion (RDM) surround (Wilke et al. 2003). Previous work employing GFS performed at rest has shown that parieto‐occipital alpha amplitudes in the second prior to the motion onset were significantly decreased preceding target disappearances, compared to when the target remained visible (Poland et al. 2021). Therefore, in the current study, exercise‐induced changes in alpha oscillations may serve not only as an index of cortical excitability, but may also predict perceptual suppression.

How might perceptual suppression and related pre‐RDM parieto‐occipital alpha activity be altered during cycling? Evidence from prior studies suggests that alpha activity often increases during exercise (Hosang et al. 2022; Bullock et al. 2017; Hottenrott et al. 2013; Enders et al. 2016; Bailey et al. 2008; Fumoto et al. 2010), and extending the baroreceptor hypothesis to the exercise context further links elevated heart rates during exercise to decreased cortical excitability and increased alpha oscillations (Crabbe and Dishman 2004; Hosang et al. 2022; Koriath et al. 1987; Lacey and Lacey 1978). However, decreases in alpha activity during physical activity have also been reported in some studies (Cao and Händel 2019; Storzer et al. 2016), presenting an alternative possibility. Based on these observations and previous GFS findings (Poland et al. 2021), we hypothesized that decreased pre‐RDM (i.e., pre‐random dot motion) parieto‐occipital alpha amplitudes would be associated with higher subjective target suppression rates, whereas increased pre‐RDM parieto‐occipital alpha amplitudes would correspond to higher target visibility rates.

We also investigated how cycling modulates HEPs. Given the heightened exteroceptive attention required to perform a perceptual and motor task simultaneously, we hypothesized that interoceptive processing would be reduced, reflected by decreased HEP amplitudes during cycling. Additionally, we explored if HEPs predict perceptual suppression. Given that perceptual suppression is associated with lower pre‐RDM occipital alpha activity, which indicates increased external attention (Kelly et al. 2006; Thut et al. 2006; Sauseng et al. 2005), increased exteroceptive attention might be correlated with target suppression during GFS. If increased exteroceptive processing competes with interoceptive processing (Skora et al. 2022), we might expect lower HEP amplitudes when the target disappears.

## 2 Methods

### 2.1 Participants

Forty‐nine healthy volunteers, mostly university students, were initially recruited for the current study. To be eligible for participation, individuals were required to have no history of neurological, psychological, cardiovascular, respiratory, metabolic, endocrine, immune, or substance abuse disorders. Further eligibility criteria included not taking regular medications that might alter cardiovascular, autonomic, or cognitive functioning, not being pregnant, and not concurrently participating in pharmacological or stimulation studies. All subjects were also required to have normal or corrected‐to‐normal vision. Of the recruited participants, two did not complete all sessions of the experiment. During the practice session on Day 1, five participants never perceived the target disappearing, and two perceived it disappearing in every trial; all seven were excluded from further participation in the study on Day 2. Ten subjects were excluded from further analysis due to noisy EEG data, largely caused by movement artifacts during the cycling conditions, as their recordings required more than 15% of channels to be interpolated. The final cohort consisted of 30 subjects (14 male, 16 female; mean age ± SD: 23.93 ± 2.41 years, age range: 20–29 years). One subject was excluded from the HEP analysis due to a lack of trials that met the inclusion criteria for this analysis in one condition. The study was conducted in accordance with the ethical guidelines of the Declaration of Helsinki. The experimental procedures were approved by the ethics committee of the University Medicine Göttingen (UMG, Germany). All subjects gave written informed consent before the study and were paid for their participation.

### 2.2 Stimuli and Task

**FIGURE 1:**
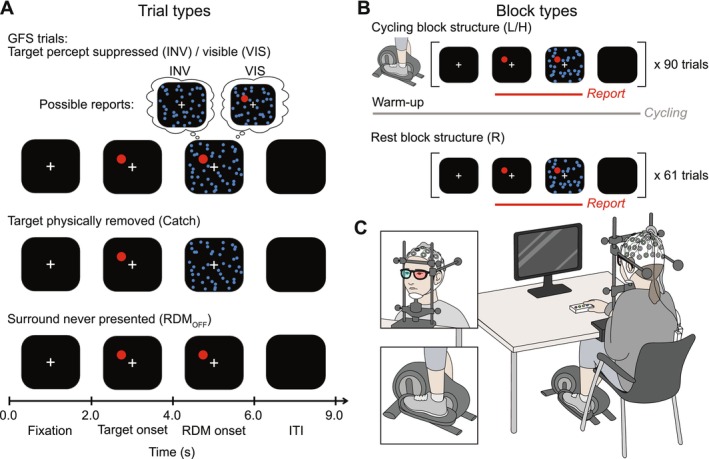
GFS trial types, block design, experimental setup. (A) Time courses of GFS, catch, and RDM_OFF_ trials. GFS trials began with a 2 s central fixation, followed by the onset of a salient red target in the upper left visual hemifield. After 2 s of target presentation, a random dot motion (RDM) stimulus was presented for a further 2 s, resulting in the disappearance of the target in a subset of trials. During catch trials, the target was physically removed at RDM onset. During RDM_OFF_ trials, the RDM pattern was never presented. Subjects reported their detection of the target by pressing and holding the button for as long as they could see it, and releasing the button to indicate its disappearance. A blank screen was presented for 3 s as the inter‐trial interval (ITI). (B) The three rest (R) blocks each consisted of 61 trials: 44 GFS, 10 catch, and 7 RDM_OFF_. The four cycling blocks (2 low‐resistance (L) and 2 high‐resistance (H)) each consisted of 90 trials: 65 GFS, 15 catch, and 10 RDM_OFF_. Each cycling block began with a 3‐min warm‐up period before the GFS paradigm was presented. (C) The experimental setup consisted of the subject seated at a desk facing a computer screen where the perceptual task was presented. They reported their perceptual responses using a button box placed on the desk. They wore red‐blue anaglyphic glasses, and their head was positioned on a chin rest, stabilized by positioning supports on either side and against their forehead (top inset). During the cycling blocks, they cycled using an ergometer placed under the desk (bottom inset). EEG and ECG were recorded simultaneously.

The visual stimuli were programmed in MATLAB R2015b (The MathWorks Inc., Natick, MA, USA) using Psychtoolbox‐3 (Brainard 1997; Kleiner et al. 2007). Each trial began with a white fixation cross on a black background that remained visible throughout the trial (Figure 1A). After 2 s of central fixation, the target, a red disk with a diameter of 3° of visual angle, was presented in the left visual hemifield, positioned 7° horizontally and 3° vertically from the center. After an additional 2 s, a random dot motion (RDM) pattern of moving blue dots (dot diameter = 0.08°; dot speed = 10°/s; dot density = 1.0 dot/deg^2^) appeared for 2 s. A 0.5° buffer zone, set to the background color, was maintained between the target and the RDM pattern. The target and RDM pattern were each presented monocularly using red‐blue anaglyphic glasses. In a subset of trials, the presentation of the RDM stimulus resulted in the subjective disappearance of the target. Following each trial, a blank screen was shown for 3 s as the inter‐trial interval (ITI).

Subjects were instructed to maintain fixation on the fixation cross throughout the trial and to use a button box (4 Button Curve Right; Current Designs Inc., Philadelphia, PA, USA) to report their perception of the target. Using the index finger of their dominant hand (27 right‐handed, 3 left‐handed as assessed by the short version of the Edinburgh Handedness Inventory (Oldfield 1971)), participants pressed the response button upon target appearance. They were instructed to hold the button down as long as the target was visible, releasing it if the target disappeared. If the target reappeared before the end of the trial, they were asked to report this by pressing and holding the button again.

Two types of control trials (Figure 1A) were intermixed with the experimental trials: catch trials and RDM_OFF_ trials. In the catch trials, the target was physically removed upon the onset of the RDM pattern, while in the RDM_OFF_ trials, the RDM pattern was never presented.

The subjects performed GFS under three exercise intensity conditions (Figure 1B): at rest (R), and during low‐resistance (L) or high‐resistance cycling (H). The two cycling conditions were performed using an ergometer (Sportstech DFX100; Sportstech, Berlin, Germany) placed under the desk. According to the American College of Sports Medicine guidelines (Garber et al. 2011) on exercise intensity, categorized by percentage of maximal heart rate (HRmax, estimated by the formula 211 − 0.64 × age) (Nes et al. 2013), the low‐resistance condition would be classified as very light (< 50% HRmax), while the high‐resistance condition would be classified as light (50%–60% HRmax) intensity.

Each rest block (Figure 1B) comprised 61 trials (44 GFS, 10 catch, and 7 RDM_OFF_) and lasted approximately 12 min. Each cycling block (Figure 1B) comprised 90 trials (65 GFS, 15 catch, and 10 RDM_OFF_) and lasted a total of 15 min, beginning with a 3‐min warm‐up period without a perceptual task, followed by 12 min of simultaneous cycling and perceptual task performance. During the initial 3‐min warm‐up period for each cycling block, participants were instructed to establish a cadence of 60 rotations per minute (rpm), guided by an on‐screen clock displaying seconds to help them match the pace. When the clock was replaced by the perceptual task, participants were instructed to maintain this cadence of 60 rpm to the best of their abilities until the end of the block.

### 2.3 Experimental Procedure

The study was conducted over two sessions, spaced no more than 10 days apart. Overall, the first session lasted approximately 1–1.5 h, and the second session lasted 3.5–4 h including electrode preparation. In Session 1, participants completed a demographic survey, a questionnaire to assess interoceptive awareness (MAIA) (Mehling et al. 2012), and the Ishihara color vision test (Clark 1924). For inclusion in the study, subjects were required to correctly identify at least 19 out of 20 Ishihara plates. Following this, they received instructions for the task and completed one practice block of each of the three exercise intensity conditions.

Participants were instructed to abstain from caffeine, alcohol, or other mind‐altering substances for 3–4 h prior to Session 2, and from heavy alcohol consumption for 24 h before the session. In this session, EEG and ECG data were recorded simultaneously. Respiration data was recorded using a respiration belt measuring thoracic/abdominal movements (Respiration Belt MR; Brain Products GmbH, Gilching, Germany), but was not analyzed in the context of this paper due to motion artifacts. Following EEG and ECG preparation, an 8‐min baseline recording with eyes closed was taken, with blood pressure measured at the 7‐min mark. Participants were then reminded of the task instructions and proceeded to perform the experiment.

To perform the experimental task (Figure 1C), subjects were seated in front of a 60 × 34 cm computer screen with a resolution of 1920 × 1080 pixels and a 60 Hz refresh rate (BenQ XL2411T; BenQ, Taipei, Taiwan), and the eye‐to‐screen distance was 70 cm. They placed their head on a chin rest, and it was stabilized by positioning supports on each side of the head and a pad to lean their forehead against (HeadLock Ultra Precision Head Positioner; Arrington Research, Scottsdale, AZ, USA), to minimize head movement. The lights in the recording room were turned off during the experiment, and additional curtains were used to protect the subjects from extraneous light.

The experiment consisted of seven GFS blocks, with cycling (low‐resistance—L, high‐resistance—H) and rest (R) blocks interleaved in one of two sequence orders, either L‐R‐H‐R‐L‐R‐H or H‐R‐L‐R‐H‐R‐L, pseudorandomly assigned for each participant. Subjects were allowed adequate breaks between blocks, and special care was taken to allow heart rates to return to baseline after each cycling block. Baseline heart rate was defined as the rate observed during the initial baseline recording and blood pressure measurement. Live heart rate was monitored throughout the breaks using an armband (TikrFit; Wahoo Fitness, Atlanta, GA, USA). After the experimental blocks, another 8‐min baseline recording with eyes closed was taken.

### 2.4 EEG Acquisition and Preprocessing

EEG activity was recorded from 64 electrodes distributed over the head according to the International 10–20 system (actiCap Snap, BrainAmp MR, BrainVision Recorder; Brain Products GmbH, Gilching, Germany). Electrode impedances were kept below 20 kΩ throughout the experiment. The data were recorded at a sampling rate of 1000 Hz.

EEG data were preprocessed and analyzed using the FieldTrip toolbox (Oostenveld et al. 2011) and custom‐written software in MATLAB R2015b (The MathWorks Inc., Natick, MA, USA). The data were downsampled to 256 Hz and bandpass filtered between 0.5 and 110 Hz. A notch filter was applied to remove 50 Hz line noise. The continuous data were then segmented into trials. Trials containing muscle artifacts, jumps, or clipping artifacts were identified automatically, visually inspected, and then rejected when necessary. Overall, 18% of trials were excluded. An independent component analysis (ICA) was performed to identify eye movement‐related artifacts, and the relevant components were removed. The data were then re‐referenced to a common average reference. If there were noisy channels that required interpolation, they were removed prior to re‐referencing. They were then interpolated after the data were re‐referenced.

### 2.5 ECG Acquisition, Preprocessing, and Instantaneous Heart Rate Analysis

The ECG montage used in this study was based on the one described by Petzschner et al. (2019). Two ECG signals were acquired using two electrodes placed on the left and right clavicle (active electrodes), two electrodes placed at the left and the right hip/abdominal regions (reference electrodes), and a ground electrode placed between the shoulder blades (BrainAmp ExG; Brain Products GmbH, Gilching, Germany). The second ECG (left clavicle—right hip) served as a backup in case the signal quality of the first ECG (right clavicle—left hip) was too low for reliable R‐peak or T‐wave detection. In the current dataset, the first ECG signal was of high quality for all but one participant, for whom the second ECG was used in the analysis.

R‐peaks were identified using the Pan and Tompkins algorithm (Pan and Tompkins 1985), using code custom‐written in MATLAB R2015b (The MathWorks Inc., Natick, MA, USA), combined with code modified from Sedghamiz (2014). The peaks of the P wave, Q wave, S wave, and T wave were identified using a custom‐written code implementing the method proposed in Leutheuser et al., for the detection of these four fiducial points (Leutheuser et al. 2016). The end of the T wave was determined using a custom‐written code implementing a trapezoidal area algorithm (Vázquez‐Seisdedos et al. 2011), and this method was also adapted for the detection of the beginning of the P wave. The pre‐RDM instantaneous heart rate was obtained by dividing 60 by the inter‐beat interval (distance between two R‐peaks) preceding RDM onset. Heart rate variability (HRV) during the pre‐RDM period was quantified using the root mean square of successive differences (RMSSD), a commonly used time‐domain method that reflects short‐term parasympathetic activity. RMSSD was calculated over the whole 4‐s pre‐RDM period using the formula:

$$\text{RMSSD}=\sqrt{\frac{1}{N-1}\sum_{i+1}^{N-1}{\left({\mathrm{RR}}_{i+1}-{\mathrm{RR}}_{i}\right)}^{2}}$$

where ${\mathrm{RR}}_{i}$ represents the i‐th interbeat interval, and N is the total number of intervals within that time window.

### 2.6 Behavioral Analysis

The behavioral analyses were performed using custom‐written scripts in MATLAB R2015b (The MathWorks Inc., Natick, MA, USA). For each subject, the disappearance probability during GFS trials in each of the three exercise intensity conditions was calculated by dividing the number of trials with at least one reported target disappearance by the total number of GFS trials performed. The reaction time for the catch trials was defined as the average time taken after RDM onset/target removal to report the disappearance of the target. The average disappearance latencies for GFS trials were calculated by subtracting the reaction time for catch trials from the time at which the first disappearance was reported for each of the GFS trials with at least one reported target disappearance. The reappearance probability during GFS trials was calculated by dividing the number of trials with more than one reported target disappearance by the total number of GFS trials performed.

### 2.7 Alpha Amplitude Analysis

For the analysis of pre‐RDM alpha amplitudes, the data of the parieto‐occipital electrodes O1, O2, Oz, POz, PO3, PO4, PO7, PO8, and Iz during the course of the whole trial were band‐pass filtered at 8–12 Hz with a 4th‐order Butterworth filter and subsequently Hilbert transformed. The pre‐RDM alpha amplitudes were obtained by taking the absolute values of the Hilbert transform, equivalent to the envelope of the filtered signal, in a time window spanning the second prior to the onset of the RDM stimulus (pre‐RDM window), pooled over the parieto‐occipital electrodes. The data were baseline‐corrected using a baseline window of 0.5 s prior to target onset. The electrodes were chosen based on previous research that investigated pre‐RDM alpha power during GFS across the same set of electrodes (Poland et al. 2021). The analysis for the other time windows (1 s prior to target onset, 1 s following target onset, 1 s following RDM onset/post‐RDM onset, the last second of RDM onset/1 s post‐RDM onset) was conducted in a similar fashion. The 2 s between target onset and RDM onset were split into two separate windows, instead of taking the whole time period as a pre‐RDM window, to be able to distinguish between the response associated with target adaptation and the pre‐RDM preparatory processes prior to RDM onset, in other words, the neural factors that might lead to perceptual suppression. Similarly, the 2 s after RDM onset were split into two separate windows (post‐RDM onset and 1 s post‐RDM onset), to distinguish between neural responses to the RDM onset and any subsequent perceptual response registration (which predominantly took place in the first second after RDM onset), and any post‐response neural modulations.

We also report differences in pre‐RDM pre‐frontal alpha amplitude across the three exercise intensity conditions, calculated over the Fp1, Fp2, AF3, AF7, AFz, AF4, AF8 channels.

For the comparison between exercise intensity conditions (levels: rest, low‐resistance cycling, high‐resistance cycling), we analyzed the alpha amplitudes for all GFS trials for each of the three conditions retained after preprocessing. To determine each subject's individual alpha frequency (IAF), we performed a Fast Fourier Transform (FFT) on the 1 s pre‐RDM time window for all parieto‐occipital electrodes for each exercise intensity condition, analyzing frequencies between 1 and 30 Hz with a resolution of 1 Hz. We then identified the peak frequency within the 8–12 Hz range for each subject. For the topographical representations, we calculated the average absolute value of the 8–12 Hz filtered Hilbert transform during the second prior to RDM onset for each channel individually, then subtracted the rest condition values from those of the cycling conditions.

For the two‐factor comparison of target visibility (levels: visible, invisible) and exercise intensity, we analyzed the alpha amplitudes separately for trials in which subjects reported the target's disappearance and trials in which that target was reported to have remained visible, across each of the three exercise intensity conditions.

### 2.8 Heartbeat‐Evoked Potentials Analysis

Heartbeat‐evoked potentials (HEPs) were computed on EEG signals locked to the T‐peak of the ECG. Only trials with T‐peaks occurring at least 300 ms after target onset to 400 ms before RDM onset were chosen to avoid the HEPs from being contaminated by responses to the target or the RDM onset. The EEG signals were segmented from 1000 ms before the T‐peak to 2000 ms after the T‐peak and then averaged to generate the pre‐RDM T‐locked responses to heartbeats.

Nine subjects had no trials that met the inclusion criteria for this analysis in the high‐resistance condition. Additionally, eight subjects (not mutually exclusive from the nine previously mentioned) had heart rates in the high‐resistance condition that were too high (R‐to‐R interval < 550 ms) to allow for a sufficient time window between heartbeats for statistical analysis. Consequently, the high‐resistance condition was excluded from this analysis. One subject had no trials meeting the inclusion criteria for this analysis in the low‐resistance condition and was therefore also excluded from this analysis, resulting in a sample size of 29.

For the HEP analysis, the significantly different heart rates between the two exercise intensity conditions (rest vs. low‐resistance) were taken into account when selecting the statistical window. To account for variations in the timing of T‐waves and P‐waves and their respective cardiac field artifacts, the ends of the T‐waves and the beginnings of the subsequent P‐waves were carefully inspected on each trial. Based on this inspection, a 133–234 ms post‐T‐peak time window was chosen to ensure HEPs submitted for further statistical analysis are free from cardiac electrical artifacts.

For the analysis of the effect of exercise intensity on HEPs, the selected window and all electrodes were submitted to a cluster‐based permutation *t*‐test (Maris and Oostenveld 2007) as implemented in the FieldTrip toolbox (Oostenveld et al. 2011). This test compared the HEP amplitudes between conditions (rest vs. low‐resistance), identifying clusters of significant differences across electrodes and time points. The Monte Carlo method was used to generate the permutation distribution by randomly shuffling the condition labels 5000 times to calculate cluster‐level statistics. Clusters were formed based on a *t*‐statistic threshold of *p* < 0.05, with adjacent electrodes considered neighbors; a minimum of two neighboring channels was required for a cluster to be considered. Significant differences in clusters were identified if the cluster‐level *p* value, corrected using the maximum cluster sum statistic, was below 0.05.

To test for a 2 × 2 interaction effect of target visibility (visible vs. invisible) and exercise intensity (rest vs. low‐resistance) on HEP amplitudes, a similar procedure was adopted, but a repeated measures permutation *F*‐test was employed instead of a *t*‐test. This analysis compared the target visibility effect at rest (Rest (visible‐invisible)) against the target visibility effect at low‐resistance (Low‐resistance (visible‐invisible)) to assess whether the target visibility effect differed significantly between exercise intensity conditions.

For any significant clusters identified by the cluster‐based permutation tests, the average HEP amplitudes over the electrodes and time points within the cluster were extracted. These average amplitudes were then submitted to paired samples *t*‐tests to further validate the observed differences between conditions.

#### 2.8.1 Control Analyses for Possible Effects of Cardiovascular Artifacts

HEPs represent neural responses to cardiac signals but can also include cardiac field artifacts and pulse‐related artifacts (Kern et al. 2013). One commonly used approach to mitigate any associated volume conduction effects of these artifacts is independent component analysis (ICA). However, this approach has been criticized for its limited ability to completely eliminate the cardiac field artifacts and for the potential risk of removing relevant task‐related signals (Petzschner et al. 2019). To ensure that the observed effects in HEP amplitudes were not due to differences in cardiac electrical activity directly affecting EEG data by volume conduction, we submitted the mean ECG amplitudes to the same cluster‐based permutation tests as the observed effects.

Several studies have also controlled for heart rate when differences in HEPs were observed (Park and Blanke 2019; Coll et al. 2021). By using exercise as an intervention, the rest and low‐resistance conditions have significantly different heart rates by design. We thus fitted a linear mixed‐effect model (LME) to investigate the effects of exercise intensity conditions and heart rate on HEP amplitude. The model examined the main effects of exercise intensity condition and heart rate on HEP amplitude, with condition and heart rate as fixed effects and subject included as a random intercept to account for repeated measures within participants. Assumptions of linearity, homoscedasticity, and no multicollinearity were evaluated prior to model fitting, while the normality of residuals was assessed *post hoc*. Model fitting and parameter estimation were conducted using maximum likelihood estimation in MATLAB R2015b (The MathWorks Inc., Natick, MA, USA). Model fit was assessed using the Akaike Information Criterion (AIC), Bayesian Information Criterion (BIC), and log‐likelihood values, with statistical significance set at *α* = 0.05.

### 2.9 Statistical Analyses

Statistical analyses were conducted in SPSS Statistics for Windows, Version 27.0 (IBM Corp., Armonk, NY, USA). Data were tested for the normality assumption using the Shapiro–Wilk test.

When testing for the effects of exercise intensity (3 levels: rest, low‐resistance cycling, high‐resistance cycling), data that met the normality assumption were analyzed using repeated‐measures ANOVA (RM‐ANOVA), followed by Bonferroni‐corrected *post hoc* pairwise comparisons when significant effects were found. When testing for the effects of target visibility and exercise intensity, if normality was satisfied, a 2 (factor: target visibility; levels: visible, invisible) × 3 (factor: exercise intensity; levels: rest, low‐resistance cycling, high‐resistance cycling) repeated‐measures ANOVA was applied. Mauchly's test was used to assess sphericity, and if this assumption was violated, the Greenhouse–Geisser correction was applied. If the test revealed significant effects, *post hoc* Bonferroni‐corrected pairwise comparisons were performed.

Data that did not satisfy the normality assumption were analyzed using the Friedman test to assess differences across conditions. When the Friedman test indicated a significant effect, *post hoc* pairwise comparisons were conducted using the Wilcoxon signed‐rank test to identify specific differences between conditions, with a Bonferroni correction applied for multiple comparisons. For the analysis of the effects of exercise intensity and target visibility on alpha amplitudes, a Bonferroni‐Holm correction was applied due to the larger number of comparisons in this analysis.

Partial eta‐squared ($\eta_{p}^{2}$), Cohen's *d*, Kendall's *W*, and rank‐biserial correlation (*r*) were calculated as the effect sizes for the repeated‐measures ANOVAs, *t*‐tests, Friedman tests, and Wilcoxon signed‐rank tests, respectively.

For comparisons against zero, a one‐sample *t*‐test was used for data meeting the normality assumption, while a Wilcoxon signed‐rank test was applied for data that did not meet this assumption.

For correlation analyses, Pearson correlation was employed for normally distributed data, while Kendall's Tau‐b was applied for data that did not meet the normality assumption.

## 3 Results

### 3.1 Behavioral Analysis

**FIGURE 2:**
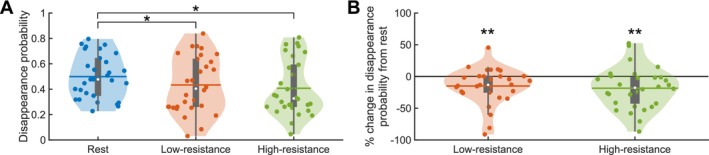
Disappearance probability decreases for cycling conditions compared to rest. (A) Violin plots showing the mean disappearance probabilities during GFS performed at rest (blue), low‐resistance (orange), and high‐resistance (green) cycling. Statistical significance between the conditions, as assessed by *post hoc* Bonferroni‐corrected pairwise comparisons, is indicated at the *p* ≤ 0.05 (*) level. (B) Violin plots showing the percent change in disappearance probability for low‐resistance (orange) and high‐resistance (green) cycling compared to rest. Statistical significance, as assessed by one‐sample *t*‐tests, is indicated at the *p* ≤ 0.01 (**) level. For both panels, vertical gray boxes indicate the interquartile range (IQR), with gray whiskers indicating 1.5 × IQR, surrounded on each side by the kernel density estimation in the color corresponding to each group. Horizontal lines, in the color corresponding to each group, denote means, and white dots indicate medians. Each dot in a given condition represents the average value of a single subject. *N* = 30 for all plots.

Subjects performed a Generalized Flash Suppression (GFS) task under three exercise intensity conditions: at rest, and during low‐resistance or high‐resistance cycling. In the GFS task, a salient target stimulus is typically completely suppressed, that is, rendered subjectively invisible, after the onset of a random dot motion (RDM) stimulus (see Section 2.2—*Stimuli and task*). We tested whether there is an effect of exercise on the probability of target suppression during GFS trials (Figure 2A). The RM‐ANOVA revealed an effect of exercise intensity on disappearance (i.e., target suppression) probability (*F*(2, 58) = 6.40, *p* = 3.08 × 10^−3^, $\eta_{p}^{2}$ = 0.18). *Post hoc* Bonferroni‐corrected pairwise comparisons showed that the disappearance probability was lower for the low‐resistance (mean ± SEM: 0.43 ± 0.04; *p* = 0.02) and high‐resistance (mean ± SEM: 0.41 ± 0.04; *p* = 0.01) cycling conditions compared with rest (mean ± SEM: 0.50 ± 0.03), but there was no difference between the two cycling conditions (*p* = 1.00). On average, this corresponded to a 14.99% decrease in disappearance probability for the low‐resistance condition (one‐sample *t‐*test, *t*(29) = −2.92, *p* = 6.78 × 10^−3^, Cohen's *d* = −0.53) and an 18.45% decrease for the high‐resistance condition (one‐sample *t‐*test, *t*(29) = −3.15, *p* = 3.74 × 10^−3^, Cohen's *d* = −0.58) compared to rest (Figure 2B).

No significant differences between the three exercise intensity conditions were observed in the percentage of correct reports during both RDM_OFF_ trials (Friedman test, $\chi^{2}$(2) = 0.19, *p* = 0.91, Kendall's W = 3.11 × 10^−3^; overall mean ± SD: 96.52% ± 4.98%; median: 100%) and catch trials (Friedman test, $\chi^{2}$(2) = 3.08, *p* = 0.22, Kendall's W = 0.05; overall mean ± SD: 97.32% ± 4.90%; median: 100%). The high detection rates across these control trials suggest subjects' percept detection and reporting abilities remained unimpaired across the exercise intensity conditions.

### 3.2 Alpha Amplitude Analysis

**FIGURE 3:**
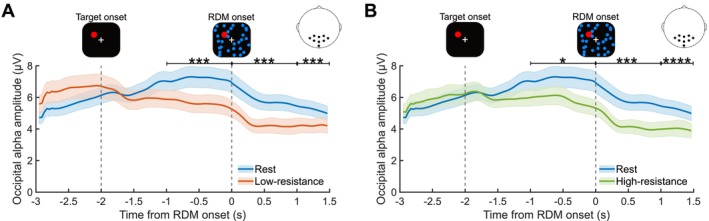
Decreased parieto‐occipital alpha amplitude during cycling compared to rest. Average time courses of alpha band (8–12 Hz) amplitudes for GFS performed during (A) low‐resistance (orange) and (B) high‐resistance (green) cycling compared to rest (blue) (*N* = 30). The shaded areas denote the SEM for each time course. The zero mark denotes the onset of the RDM stimulus, after which the target was potentially perceptually suppressed. The data represent the mean of all parieto‐occipital electrodes. Statistical significance between the conditions for different time windows, as assessed by Bonferroni‐corrected Wilcoxon signed‐rank tests, is indicated at the *p* ≤ 0.05 (*), *p* ≤ 0.001 (***), and *p* ≤ 0.0001 (****) levels.

**FIGURE 4:**
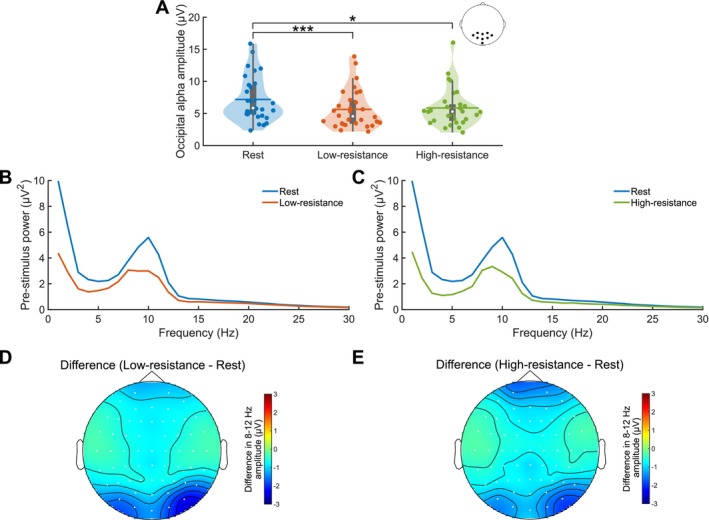
Decreased pre‐RDM parieto‐occipital alpha power for cycling conditions compared to rest. (A) Violin plots showing the mean pre‐RDM parieto‐occipital alpha during GFS performed at rest (blue), low‐resistance (orange), and high‐resistance (green) cycling. Vertical gray boxes indicate the interquartile range (IQR), with gray whiskers indicating 1.5 × IQR, surrounded on each side by the kernel density estimation in the color corresponding to each group. Horizontal lines, in the color corresponding to each group, denote means, and white dots indicate medians. Each dot in a given condition represents the average value of a single subject. Statistical significance between conditions, as assessed by Bonferroni‐corrected Wilcoxon signed‐rank tests, is indicated at the *p* ≤ 0.05 (*) and *p* ≤ 0.001 (***) levels. Power spectra for the second preceding the RDM stimulus for GFS performed at rest and during (B) low‐resistance and (C) high‐resistance cycling across subjects. Topographies of the (D) low‐resistance − rest and (E) high‐resistance − rest differences in 8–12 Hz amplitude in the second prior to RDM onset. *N* = 30 for all plots.

Given that cycling modulates alpha activity (8–12 Hz) (Crabbe and Dishman 2004; Hosang et al. 2022) and pre‐RDM parieto‐occipital alpha amplitudes are predictive of perceptual suppression (Poland et al. 2021), we analyzed the effect of exercise intensity on parieto‐occipital alpha amplitudes during the second preceding RDM onset. There was a statistically significant difference in the mean parieto‐occipital alpha amplitude over this time window (Figure 3 and Figure 4A) between exercise intensity conditions (Friedman test, $\chi^{2}$(2) = 16.80, *p* = 2.25 × 10^−4^, Kendall's W = 0.29). Parieto‐occipital alpha amplitudes were lower during cycling compared to rest (Bonferroni‐corrected Wilcoxon signed‐rank tests, low‐resistance: *Z =* −3.86, *p* = 3.45 × 10^−4^, *r* = 0.50; high‐resistance: *Z =* −2.89, *p* = 0.01, *r* = 0.37), but there was no difference between the two cycling conditions (*Z =* −0.85, *p* = 1.18, *r* = 0.11).

We also found differences in parieto‐occipital alpha amplitudes due to exercise intensity in other time windows (Figure 3); specifically, during the first second of RDM presentation (post‐RDM onset; Friedman test, $\chi^{2}$(2) = 29.07, *p* = 4.88 × 10^−7^, Kendall's W = 0.48) and the subsequent half‐second (1 s post‐RDM onset, Friedman test, $\chi^{2}$(2) = 26.87, *p* = 1.47 × 10^−6^, Kendall's W = 0.45). *Post hoc* Bonferroni‐corrected Wilcoxon signed‐rank tests revealed a significant decrease in parieto‐occipital alpha amplitude during cycling, compared with rest, also in both later time windows (post‐RDM onset results: low‐resistance, *Z =* −3.92, *p* = 2.68 × 10^−4^, *r* = 0.50; high‐resistance, *Z =* −3.98, *p* = 2.07 × 10^−4^, *r* = 0.51; 1 s post‐RDM onset results: low‐resistance, *Z =* −3.63, *p* = 8.49 × 10^−4^, *r* = 0.47; high‐resistance, *Z =* −4.19, *p* = 8.53 × 10^−4^, *r* = 0.54). No significant differences were observed between the two cycling conditions (post‐RDM onset results: *Z =* −0.09, *p* = 2.78, *r* = 0.01; 1 s post‐RDM onset results: *Z =* −0.92, *p* = 0.36, *r* = 0.12).

The power spectra of the second prior to RDM onset revealed that the peak individual alpha frequency (IAF) did not differ across the three exercise intensity conditions (Friedman test, $\chi^{2}$(2) = 5.26, *p* = 0.07, Kendall's W = 0.09), and the mean IAF across subjects was 9.62 Hz ± SD 1.05 Hz. However, the cycling conditions led to a downshift of the EEG power spectrum compared to rest, and an analysis of the power at peak IAF (Figure 4B,C) revealed a difference between the three conditions (Friedman test, $\chi^{2}$(2) = 15.27, *p* = 4.84 × 10^−4^, Kendall's W = 0.25). Corroborating the pre‐RDM, cycling‐induced decrease in alpha amplitude, decreased power was observed for low‐resistance (Bonferroni‐corrected Wilcoxon signed‐rank test, *Z =* −3.67, *p* = 7.24 × 10^−4^, *r* = 0.47) and high‐resistance (*Z =* −2.77, *p* = 0.02, *r* = 0.36) cycling compared to rest, but there was no difference in power between the cycling conditions (*Z =* −0.36, *p* = 2.16, *r* = 0.05). The topographies of the cycling—rest difference in alpha amplitude (Figure 4D,E) suggested that the decrease in alpha activity was most prominent in the parieto‐occipital cortex.

In addition to parieto‐occipital alpha amplitudes, we examined pre‐RDM prefrontal alpha amplitudes, since exercise has been reported to elicit prominent effects in the alpha band in anterior regions (Hosang et al. 2022). A significant effect of exercise intensity on pre‐RDM prefrontal alpha was observed (Friedman test, $\chi^{2}$(2) = 18.07, *p* = 1.19 × 10^−4^, Kendall's W = 0.30). However, *post hoc* Bonferroni‐corrected Wilcoxon signed‐rank tests revealed that both low‐resistance (*Z* = −4.10, *p* = 1.22 × 10^−4^, *r* = 0.75) and high‐resistance (*Z* = −3.65, *p* = 7.84 × 10^−4^, *r* = 0.67) cycling conditions resulted in significantly *lower* prefrontal alpha amplitudes compared to rest. No significant difference was found between the two cycling conditions (*Z* = −0.61, *p* = 1.63, *r* = 0.11). We also examined whether changes in prefrontal alpha amplitude correlated with changes in disappearance probability. No significant correlation was observed between the percentage change in pre‐RDM prefrontal alpha amplitude from rest and the percentage change in disappearance probability, for neither low‐resistance (Kendall's Tau‐b, $\tau_{b}$(30) = −0.05, *p* = 0.78) nor high‐resistance cycling ($\tau_{b}$(30) = −0.08, *p* = 0.56).

**FIGURE 5:**
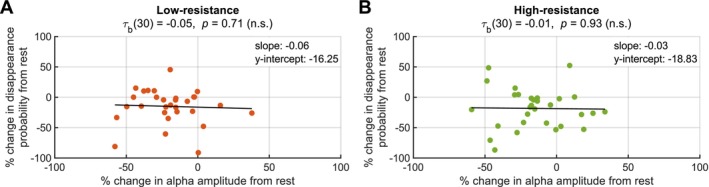
No correlation between pre‐RDM parieto‐occipital alpha amplitude and disappearance probability. Correlations, as assessed by Kendall's Tau‐b, between percent change in parieto‐occipital alpha amplitude from rest and percent change in disappearance probability from rest for (A) low‐resistance and (B) high‐resistance cycling. *N* = 30 for all plots.

In an analysis of the relationship between cycling‐related changes in alpha amplitude and changes in disappearance probability (Figure 5), we observed no statistically significant correlations for either the low‐resistance and high‐resistance cycling conditions (Kendall's Tau‐b, all *p*s > 0.05).

**FIGURE 6:**
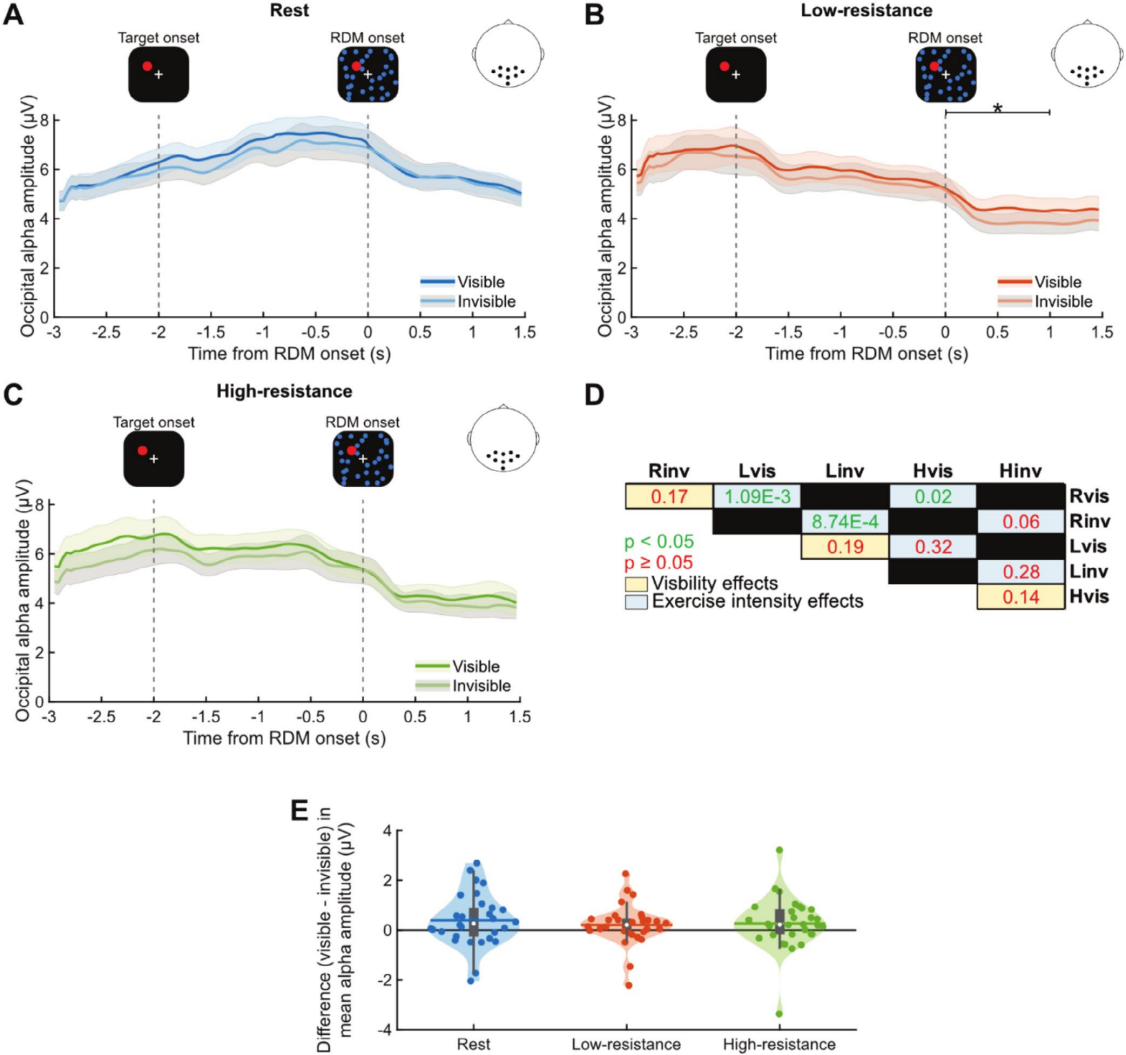
Pre‐RDM parieto‐occipital alpha amplitude as a function of perceptual suppression and cycling. Average time courses of alpha band (8–12 Hz) amplitudes for GFS trials where the target remained visible and trials where the target was rendered invisible, when the task was performed during (A) rest, (B) low‐resistance, and (C) high‐resistance cycling. The SEM is plotted for each time course in corresponding color. The zero mark denotes the onset of the RDM stimulus, after which the target was potentially perceptually suppressed. The data represent the mean of all parieto‐occipital electrodes. Statistical significance between the conditions for different time windows, as assessed by Wilcoxon signed‐rank tests, is indicated at the *p* ≤ 0.05 (*) level. (D) Bonferroni‐Holm‐corrected Wilcoxon signed‐rank test *p*‐values for pairwise comparisons reflecting the effect of target visibility and exercise intensity on parieto‐occipital alpha amplitude in the second prior to RDM onset (Rest—R, Low‐resistance—L, High‐resistance—H, Visible—vis, Invisible—inv). (E) Violin plots showing the visible—invisible difference in mean pre‐RDM alpha during GFS performed at rest (blue), and during low‐resistance (orange), and high‐resistance (green) cycling. Vertical gray boxes indicate the interquartile range (IQR), with gray whiskers indicating 1.5 × IQR, surrounded on each side by the kernel density estimation in the color corresponding to each group. Horizontal lines, in the color corresponding to each group, denote means, and white dots indicate medians. Each dot in a given condition represents the average value of a single subject. *N* = 30 for all plots.

Based on previous work on GFS performed at rest (Poland et al. 2021), we hypothesized that pre‐RDM parieto‐occipital alpha would be lower when the target becomes perceptually suppressed compared to when it remains visible. To investigate this, we assessed whether pre‐RDM, parieto‐occipital alpha activity reflects the subjective visibility of the target in GFS trials (Figure 6) and examined how this relationship interacted with the effect of cycling. A Friedman test revealed a statistically significant difference in pre‐RDM, parieto‐occipital alpha activity across the exercise intensity and target visibility conditions ($\chi^{2}$(5) = 42.74, *p* = 4.17 × 10^−8^, Kendall's W = 0.29) (Figure 6A–C). In the *post hoc* analysis, we performed nine pairwise comparisons with the Bonferroni‐Holm‐corrected Wilcoxon signed‐rank test (Figure 6D), with three comparisons testing for the effect of exercise intensity on pre‐RDM alpha amplitudes and six comparisons testing for the effect of alpha amplitudes on target visibility. This analysis suggests alpha amplitude‐linked effects of both target visibility and exercise intensity. Violin plots in Figure 6E show that, for most subjects, pre‐RDM alpha amplitude was consistently higher for trials where the target remained visible compared to when it was rendered invisible across all exercise intensity conditions, in line with the findings in Poland et al. (2021). However, the target visibility‐related effects did not survive the correction for multiple comparisons (rest: *Z* = −2.03, *p* = 0.17; low‐resistance: *Z* = −2.07, *p* = 0.19; high‐resistance: *Z* = −1.98, *p* = 0.14; Uncorrected *p* values are reported in Table S1).

As for the cycling‐related effects on pre‐RDM alpha, we observed significant reductions in alpha amplitude during visible trials for both low‐resistance (*Z =* −3.82, *p* = 1.09 × 10^−3^, *r* = 0.49) and high‐resistance cycling (*Z =* −2.95, *p* = 0.02, *r* = 0.38) compared to rest, with no significant difference between the two cycling conditions (*Z =* −1.41, *p* = 0.32, *r* = 0.18). Similarly, in the invisible trials, a significant reduction was found between rest and low‐resistance cycling (*Z =* −3.90, *p* = 8.74 × 10^−4^, *r* = 0.50), but not between rest and high‐resistance cycling (*Z =* −2.58, *p* = 0.06, *r* = 0.33), or between the two cycling conditions (*Z =* −1.08, *p* = 0.28, *r* = 0.14). These results suggest that while cycling decreases pre‐RDM alpha amplitude, this exercise‐induced modulation is not specifically linked to perceptual suppression.

### 3.3 Heart Rate Analysis

**FIGURE 7:**
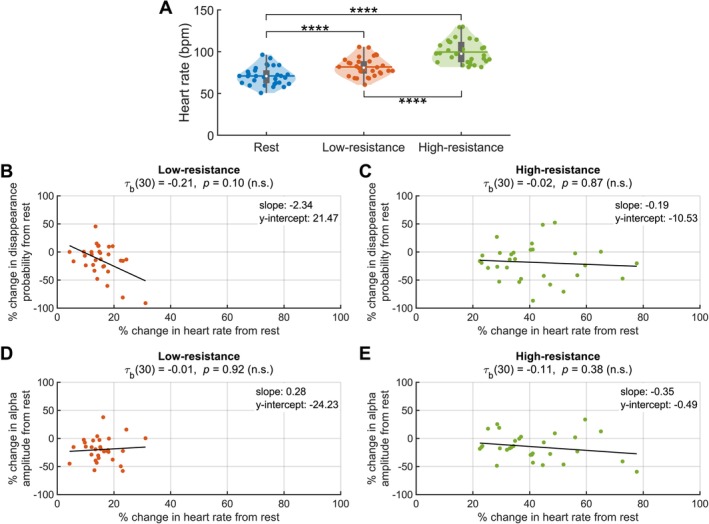
Heart rate increases with cycling intensity. (A) Violin plots showing the pre‐RDM instantaneous heart rate during GFS performed at rest (blue), low‐resistance (orange), and high‐resistance (green) cycling. Vertical gray boxes indicate the interquartile range (IQR), with gray whiskers indicating 1.5 × IQR, surrounded on each side by the kernel density estimation in the color corresponding to each group. Horizontal lines, in the color corresponding to each group, denote means, and white dots indicate medians. Each dot in a given condition represents the average value of a single subject. Statistical significance between the conditions, as assessed by a *post hoc* analysis with a Bonferroni adjustment, is indicated at the *p* ≤ 0.0001 (****) level. Correlations as assessed by Kendall's Tau‐b between percent change in heart rate from rest and percent change in disappearance probability from rest for (B) low‐resistance and (C) high‐resistance cycling. Correlations between percent change in heart rate from rest and percent change in alpha amplitude from rest for (D) low‐resistance and (E) high‐resistance cycling. None of the correlations are significant. *N* = 30 for all plots.

To investigate how the pre‐RDM instantaneous heart rate interacts with exercise intensity and conscious visual perception of the target, we conducted a two‐way repeated measures ANOVA with two factors: exercise intensity (levels: rest, low‐resistance cycling, high‐resistance cycling) and target visibility (levels: visible, invisible). The analysis revealed a significant main effect of exercise intensity (Greenhouse–Geisser corrected; *F*(1.25, 36.26) = 269.24, *p* = 1.09 × 10^−19^, $\eta_{p}^{2}$ = 0.90), confirming the efficacy of the exercise intervention. *Post hoc* Bonferroni‐corrected pairwise comparisons indicated that both low‐resistance and high‐resistance cycling elicited significantly higher heart rates compared to rest (Figure 7A; low‐resistance: 10.81 bpm difference, *p* = 2.78 × 10^−16^; high‐resistance: 28.59 bpm difference, *p* = 4.53 × 10^−17^). Additionally, high‐resistance cycling resulted in a significantly higher heart rate than low‐resistance cycling (17.77 bpm difference, *p* = 3.11 × 10^−13^). On average, the heart rate increased by 15.56% during low‐resistance cycling compared to rest, and by 41.32% during high‐resistance cycling compared to rest. However, there was no significant main effect of target visibility: the instantaneous pre‐RDM heart rate did not differ significantly between trials in which the target remained visible and those in which it disappeared (*F*(1, 29) = 0.99, *p* = 0.33, $\eta_{p}^{2}$ = 0.03). Furthermore, no significant interaction between exercise intensity and target visibility was observed (*F*(2, 58) = 0.47, *p* = 0.63, $\eta_{p}^{2}$ = 0.02).

We investigated whether these heart rate changes were associated with the cycling‐related changes in disappearance probability and pre‐RDM alpha amplitude. For the relationship between heart rate and disappearance probability, there were no statistically significant correlations for either low‐resistance or high‐resistance cycling (Figure 7B,C; Kendall's tau‐b, all *p*s > 0.05). Similarly, for the relationship between heart rate and alpha amplitude, no statistically significant correlations emerged for either condition (Figure 7D,E; Kendall's tau‐b, all *p*s > 0.05). An analysis using pre‐RDM heart rate variability (HRV), quantified with the RMSSD time‐domain method, yielded similar results. RMSSD HRV significantly decreased with increasing exercise intensity but did not vary with target disappearance/visibility and showed no significant correlations with disappearance probability or pre‐RDM alpha amplitude (Figure S1).

We also assessed phasic heart rate changes across the course of a trial, and found a deceleration in heart rate over the trial duration, which occurred irrespective of target visibility (Figure S2 and Table S2). This deceleration aligns with the well‐characterized phenomenon of cardiac deceleration following a warning stimulus, which reflects anticipation of an upcoming stimulus or response registration (Lacey and Lacey 1974, 1978; Lacey 1967; Jennings and Wood 1977; Lacey and Lacey 1980; Somsen et al. 2004; Park et al. 2014; Cobos et al. 2019; Motyka et al. 2019; Grund et al. 2022). Interestingly, in our paradigm, this phenomenon emerged despite the absence of an explicit warning stimulus, likely driven instead by the presence of stereotyped, predictable events that may have acted as implicit cues for anticipation. Furthermore, this deceleration appears to be robust, persisting even under the cycling conditions.

### 3.4 Heartbeat‐Evoked Potentials Analysis

**FIGURE 8:**
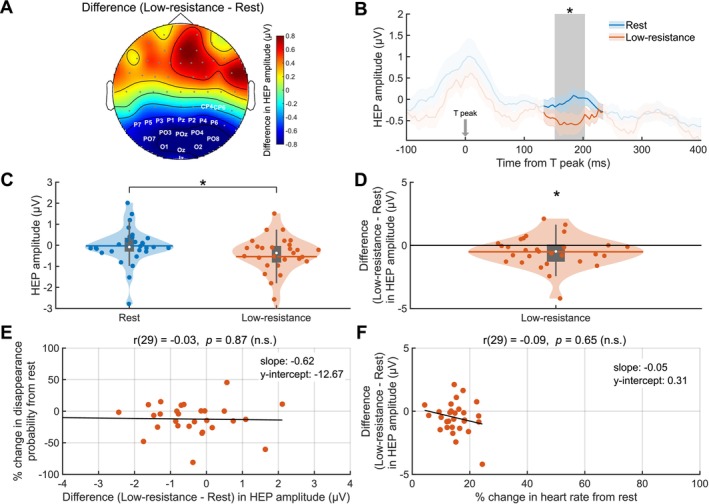
Heartbeat‐evoked potential amplitudes decrease during cycling. (A) Topographical map of heartbeat‐evoked potential (HEP) amplitude difference between low‐resistance and rest conditions in the 152–203 ms post‐T‐peak time window during which a statistically significant difference was observed. White labels indicate the channels contributing to the significant cluster. (B) Pre‐RDM HEPs for the two conditions averaged across the cluster. The shaded areas denote the SEM for each time course. The signal contaminated by T‐wave and P‐wave cardiac artifacts appears in a lighter color. The gray bar highlights the time window in which a significant difference was observed as the *p* ≤ 0.05 (*) level. (C) Violin plots of the HEP amplitude averaged across the cluster for rest (blue) and low‐resistance cycling (orange). Vertical gray boxes indicate the interquartile range (IQR), with gray whiskers indicating 1.5 × IQR, surrounded on each side by the kernel density estimation in the color corresponding to each group. Horizontal lines, in the color corresponding to each group, denote means, and white dots indicate medians. Each dot in a given condition represents the average value of a single subject. For both (B) and (C), statistical significance between the conditions was assessed by a Wilcoxon signed‐rank test and is indicated at the *p* ≤ 0.05 (*) level. (D) Difference from rest in HEP amplitudes for low‐resistance cycling. Statistical significance was assessed by one‐sample *t*‐test (*t*(28) = −2.22, *p* = 0.04, Cohen's *d* = 0.41) and is indicated at the *p* ≤ 0.05 (*) level. Correlations as assessed by Pearson correlation between (E) change in HEP amplitude and percent change in disappearance probability and (F) change in heart rate and change in HEP amplitude for cycling from rest. Neither correlation is significant. *N* = 29 for all plots.

To explore if and how neural processing of heartbeats is altered during exercise, we analyzed the effect of cycling on heartbeat‐evoked potential (HEP) amplitudes. EEG data locked to the T‐peak preceding RDM onset was averaged for the rest and low‐resistance cycling conditions. The high‐resistance cycling condition was excluded from this analysis because the heart rates were too high to allow for a sufficient time window between heartbeats for statistical analysis. For the other two conditions, a 133 to 234 ms post‐T‐peak time window, chosen to avoid overlap with the end of the previous T wave and the beginning of the subsequent P wave, ensuring HEPs are free from cardiac artifacts, was submitted to a cluster‐based permutation *t*‐test. Pre‐RDM HEPs significantly differed between rest and low‐resistance cycling (Figure 8A–C) over central, parietal, and occipital electrodes in a 152 to 203 ms post‐T‐peak time window (cluster‐level statistic = 337.85, *p* = 0.02, *N* = 29; Figure S3). The HEP amplitudes during low‐resistance cycling were lower than at rest (Wilcoxon signed‐rank test, *Z* = −2.26, *p* = 0.02, *r* = 0.42; Figure 8C,D).

The control for volume conduction, that is, comparison of the ECG waveforms for rest and low‐resistance in the same time window as the observed effect, revealed no significant differences (Figure S4). Thus, the observed differences in HEP amplitudes between the two conditions cannot be attributed to differences in cardiac electrical activity. The linear mixed‐effects model (Table S3) used to investigate the effects of exercise intensity conditions on HEP amplitude, while controlling for heart rate, revealed no significant difference in HEP amplitudes between rest and low‐resistance cycling (*b* = 0.20, *p* = 0.43). Heart rate also did not significantly predict HEP amplitude (*b* = −0.03, *p* = 0.17), though the direction of the effect suggests a possible trend toward lower HEP amplitudes at higher heart rates. Random intercepts for subject (σ = 0.39) accounted for individual variability, and the residual standard deviation was 0.84. Model fit statistics included AIC = 164.87 and BIC = 175.17. These results suggest that heart rate is an unlikely contributor to the observed difference in HEP amplitudes between conditions.

We investigated whether these modulations in HEPs were associated with cycling‐related changes in disappearance probability (Figure 8E) and found no statistically significant correlation (Pearson correlation, *r*(29) = −0.03, *p* = 0.87). We also investigated the relationship between the cycling‐related changes in heart rate and the changes in HEP amplitude (Figure 8F), and again, no statistically significant correlation was found (Pearson correlation, *r*(29) = −0.09, *p* = 0.65). A corresponding analysis using cycling‐related changes in RMSSD HRV instead of heart rate also revealed no significant correlation with changes in HEP amplitude (Figure S5).

**FIGURE 9:**
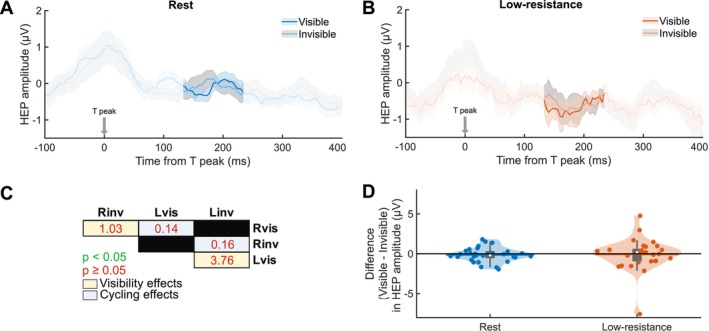
Heartbeat‐evoked potentials as a function of perceptual suppression and cycling. Pre‐RDM HEPs averaged across the significant cluster for the trials where the target remained visible and those where it was rendered invisible during GFS are shown for (A) rest and (B) low‐resistance cycling conditions. (C) Bonferroni‐corrected Wilcoxon signed‐rank test *p*‐values for pairwise comparisons reflecting the effect of target visibility and exercise on pre‐RDM HEP amplitude (Rest—R, Low‐resistance—L, Visible—vis, Invisible—inv). (D) Violin plots showing the visible—invisible difference in mean pre‐RDM HEP amplitude (from the same time window as the exercise intensity analysis) during GFS performed at rest (blue) and during low‐resistance (orange) cycling. Vertical gray boxes indicate the interquartile range (IQR), with gray whiskers indicating 1.5 × IQR, surrounded on each side by the kernel density estimation in the color corresponding to each group. Horizontal lines, in the color corresponding to each group, denote means, and white dots indicate medians. Each dot in a given condition represents the average value of a single subject. *N* = 29 for all plots.

Additionally, HEP amplitude has been linked to conscious perception of sensory stimuli at threshold (Park et al. 2014; Al et al. 2020), suggesting a role for interoceptive processing in the perception of external stimuli. To investigate this, we employed a cluster‐based permutation *F*‐test to assess the 2 × 2 interaction effect of target visibility (levels: visible, invisible) and exercise intensity (levels: rest, low‐resistance) on HEP amplitudes. This analysis revealed five positive spatio‐temporal clusters, however, neither cluster reached statistical significance (smallest cluster‐level *p* = 0.22). We also assessed whether pre‐RDM HEP amplitude reflected the subjective visibility of the target in GFS trials (Figure 9) within the same time window as the exercise intensity effect described above, as well as its interaction with the cycling effect. A Friedman test revealed a statistically significant difference in HEP amplitudes across the exercise intensity and target visibility conditions ($\chi^{2}$(3) = 8.79, *p* = 0.03, Kendall's W = 0.10). In the *post hoc* analysis, we performed four pairwise comparisons with Bonferroni‐corrected Wilcoxon signed‐rank tests (Figure 9C), with two comparisons testing for the effect of exercise intensity on pre‐RDM HEP amplitudes and two comparisons testing for the effect of pre‐RDM HEP amplitudes on target visibility. However, neither the effect of target visibility nor the effect of cycling survived the correction for multiple comparisons (all *p*s > 0.05; Uncorrected *p* values are reported in the Table S4). The difference between HEP amplitudes for trials in which the target remained visible and the trials in which it was perceptually suppressed (Figure 9D) was not significant for rest (*t*(28) = −0.83, *p* = 0.41, Cohen's *d* = −0.16) or for low‐resistance cycling (*t*(28) = −0.22, *p* = 0.83, Cohen's *d* = −0.04).

## 4 Discussion

### 4.1 Alpha Activity and Perception During Exercise

In the current study, we investigated behavioral, neural, and cardiovascular responses to Generalized Flash Suppression (GFS) performed during light‐intensity cycling. We found that the disappearance probability, that is, the rate of perceptual suppression, decreased during cycling. In parallel, pre‐RDM parieto‐occipital alpha activity was lower during cycling compared to rest. When comparing pre‐RDM alpha modulations between visible and invisible trials, we observed that parieto‐occipital alpha amplitudes were lower prior to target disappearance compared to when the target remained visible, consistently across all three exercise intensity conditions. While this difference did not reach significance after correction for multiple comparisons, the direction of the effect aligns with our previous EEG GFS study (Poland et al. 2021). These results, along with the absence of a correlation between cycling‐related modulation of alpha amplitude and disappearance probability, suggest that while pre‐RDM alpha amplitudes index target visibility, they do not account for the decreased rate of perceptual suppression during cycling.

Modulations of alpha activity have been shown to reflect fluctuations in attentional states, with the processing of attended stimuli being linked to reduced alpha activity compared to unattended stimuli (Kelly et al. 2006; Thut et al. 2006; Sauseng et al. 2005). Such reductions in alpha activity are thought to indicate heightened neural excitability (Klimesch et al. 2007; Romei, Rihs, et al. 2008; Romei, Brodbeck, et al. 2008; Jensen and Mazaheri 2010; Iemi et al. 2017) and are linked to the suppression of distracting stimuli (Kelly et al. 2006; Wöstmann et al. 2019; Foxe and Snyder 2011; Worden et al. 2000). In the previous GFS study linking alpha decreases to target disappearance at rest, it was proposed that these functions of alpha were responsible for enhancing the processing of the surrounding motion stimulus, thereby increasing its effectiveness in suppressing GFS targets (Poland et al. 2021). As in the previous study (Poland et al. 2021), we cannot distinguish between neural modulations associated with the target and the surround, which would be of interest to investigate in future studies. Understanding whether the observed alpha reductions during cycling reflect attentional shifts specific to the target, the surrounding stimulus, or a combination of both could provide further insights into the neural mechanisms underlying the reduced rate of perceptual suppression in exercise‐related contexts.

While the observed decreases in alpha activity indicate increased visual attention (Kelly et al. 2006; Thut et al. 2006; Sauseng et al. 2005), they may not be solely attributable to either target or surround processing. Instead, these reductions could reflect the dual‐task demands of dividing external attention between perceptual and motor task performance. Similar alpha modulations have been observed during walking, where reductions in parietal alpha are thought to reflect sensorimotor integration during visually guided walking in a virtual reality paradigm (Wagner et al. 2014).

Walking has been linked to more pronounced alpha power decreases than cycling, potentially reflecting the unique somatosensory and proprioceptive demands of gait (Storzer et al. 2016). Correspondingly, unique patterns of visual processing have been associated with walking and cycling. For example, studies employing orientation discrimination paradigms report different outcomes during walking and cycling. Concurrent decreases in both pre‐RDM alpha power and orientation discrimination accuracy are reported during natural walking (as opposed to stationary walking on a treadmill), with the changes in alpha suggesting a change in attentional state linked to visual awareness (Chen et al. 2022). Meanwhile, during cycling, increases in parieto‐occipital alpha relative to rest have been associated with a decrease in orientation discrimination accuracy (Bullock et al. 2017). These differing findings suggest that changes in alpha activity may vary depending on the type of exercise, potentially leading to different perceptual outcomes based on the sensory and attentional demands associated with each exercise.

### 4.2 Heart Rate and Heart Rate Variability During Exercise and Their Effect on Alpha Activity and Perception

In addition to neural and behavioral effects, our study examined exercise‐mediated cardiac influences on neural activity and perceptual outcomes in GFS. As expected, there was an increase in heart rate/decrease in heart rate variability (HRV) associated with the physical activity of cycling. However, neither the increase in heart rate nor the decrease in HRV correlated with the observed decreases in disappearance probability or pre‐RDM alpha activity. Also, none of these parameters differed significantly between trials in which the target remained visible and those in which it disappeared. The decrease in alpha observed in parallel to the increase in heart rate argues against the baroreceptor hypothesis theory of cardiovascular influences on cortical excitability during exercise (Crabbe and Dishman 2004; Hosang et al. 2022).

The absence of a correlation between heart rate and perceptual or neural outcomes also suggests that the sustained heart rate increases during cycling may reflect a general sympathetic arousal state that does not directly map onto perceptual processing. This stands in contrast to the phasic heart rate decelerations associated with attentional orienting and stimulus anticipation Lacey and (Lacey and Lacey 1974, 1978; Lacey 1967; Jennings and Wood 1977; Lacey and Lacey 1980; Somsen et al. 2004; Park et al. 2014; Cobos et al. 2019; Motyka et al. 2019; Grund et al. 2022), which were also observed in this study. It is possible that the tonic sympathetic dominance induced by exercise may override or mask the more subtle interoceptive effects that might otherwise influence perceptual suppression.

A further consideration is the interplay between respiration and cardiac function. While increased heart rate is often associated with increased baroreceptor firing due to greater cardiac output and arterial stretch, in some situations there is a context‐dependent dissociation between heart rate and baroreceptor signaling. One such case is respiratory sinus arrhythmia (RSA), the natural fluctuation in heart rate across the respiratory cycle. RSA reflects cyclic autonomic modulation aimed at optimizing pulmonary gas exchange: heart rate increases during inhalation, driven by sympathetic activation and vagal withdrawal, and decreases during exhalation due to baroreflex‐mediated vagal engagement (Noble and Hochman 2019; Berntson et al. 1993). Importantly, this means that during inhalation, heart rate is high but baroreceptor activity is relatively low. It follows that inhalation has sometimes also been associated with enhanced perception and reduced alpha power, indicating increased cortical excitability (Kluger et al. 2021), despite this being a phase of elevated heart rate and lower baroreceptor firing. This supports the idea that cortical excitability can increase even when baroreceptor activity is low, depending on the respiratory phase and overall autonomic state.

A previous study showed that RSA systematically decreases during exercise, although it can still be observed at heart rates (HR) above 100 beats per minute (Hatfield et al. 1998). Together with the observed exercise‐induced reduction of HRV, it means that exercise further complicates this relationship by reducing RSA and HRV, effectively narrowing the dynamic range of autonomic modulation. This diminished capacity for autonomic variation may limit the brain's ability to flexibly shift between interoceptive and exteroceptive modes, potentially affecting perceptual outcomes. In this sense, it may not be just the level of baroreceptor activity that matters, or the extent of parasympathetic/sympathetic dominance, but also the autonomic variability and adaptability.

### 4.3 Exercise‐Induced Effects on HEP Amplitudes

In addition to changes in heart rate and alpha activity, we also observed cycling‐related differences in HEPs, reflecting a change in the neural responses to cardiac signals during exercise. Increased HEP amplitudes have been linked to enhanced interoceptive attention, reflecting both explicit attention to (Montoya et al. 1993; Petzschner et al. 2019; Schandry et al. 1986; Pollatos and Schandry 2004; Pollatos et al. 2005; Villena‐González et al. 2017), and unconscious processing of heartbeats (Kern et al. 2013; Perogamvros et al. 2019). We observed a decrease in HEP amplitudes during cycling compared to rest, suggesting that interoceptive processing is attenuated during exercise, potentially reflecting an attentional shift away from interoceptive information and toward exteroceptive information required for the simultaneous performance of a perceptual and a motor task. Our results also indicate that the difference in heart rate between the rest and low‐resistance cycling conditions does not contribute to the observed difference in HEP amplitudes between them.

Furthermore, in our study, the HEPs did not predict perceptual suppression in GFS at rest or during cycling, nor did cycling‐induced changes in HEPs correlate with changes in disappearance probability. These results suggest that while HEPs are sensitive to changes in physiological state, their role in mediating perceptual outcomes may be limited, at least in the context of GFS. This contrasts with preliminary evidence from previous research suggesting that increased HEP amplitudes were associated with increased detection of a target in a visual threshold paradigm (Park et al. 2014). The absence of such a link in our study suggests that the influence of interoceptive processing as indexed by HEPs on perceptual processing may vary depending on the perceptual task, highlighting the need for further investigation into the contribution of HEPs to conscious perception.

The observed exercise‐linked reductions in perceptual suppression, alpha activity, and HEP amplitude highlight a complex interplay between interoception and exteroception during physical activity. While exercise appears to enhance exteroceptive processing, as reflected in reduced perceptual suppression and reduced alpha amplitudes, it simultaneously attenuates cardiac interoceptive processing, as indexed by reduced HEPs. These findings support the idea of a dynamic trade‐off between interoceptive and exteroceptive domains, modulated by task demands and physiological state.

## Author Contributions

**Aishwarya Bhonsle:** conceptualization, data curation, formal analysis, investigation, methodology, software, writing – original draft, writing – review and editing, visualization. **Melanie Wilke:** conceptualization, methodology, resources, supervision, writing – original draft, writing – review and editing, funding acquisition.

## Conflicts of Interest

The authors declare no conflicts of interest.

## Supporting information


**Data S1:** Supporting Information.

## Data Availability

The data that support the findings of this study are available on request from the corresponding author. The data are not publicly available due to privacy or ethical restrictions.

## References

[psyp70144-bib-0001] Al, E. , F. Iliopoulos , N. Forschack , et al. 2020. “Heart–Brain Interactions Shape Somatosensory Perception and Evoked Potentials.” Proceedings of the National Academy of Sciences 117: 10575–10584. 10.1073/pnas.1915629117 PMC7229654 32341167

[psyp70144-bib-0002] Bailey, S. P. , E. E. Hall , S. E. Folger , and P. C. Miller . 2008. “Changes in EEG During Graded Exercise on a Recumbent Cycle Ergometer.” Journal of Sports Science and Medicine 7: 505–511. 24149958 PMC3761919

[psyp70144-bib-0003] Berntson, G. G. , J. T. Cacioppo , and K. S. Quigley . 1993. “Respiratory Sinus Arrhythmia: Autonomic Origins, Physiological Mechanisms, and Psychophysiological Implications.” Psychophysiology 30: 183–196. 8434081 10.1111/j.1469-8986.1993.tb01731.x

[psyp70144-bib-0004] Berntson, G. G. , K. S. Quigley , and D. Lozano . 2007. “Cardiovascular Psychophysiology.” In Handbook of Psychophysiology, edited by G. Berntson , J. T. Cacioppo , and L. G. Tassinary , 182–210. Cambridge University Press.

[psyp70144-bib-0005] Birznieks, I. , T. W. Boonstra , and V. G. Macefield . 2012. “Modulation of Human Muscle Spindle Discharge by Arterial Pulsations ‐ Functional Effects and Consequences.” PLoS One 7: e35091. 22529975 10.1371/journal.pone.0035091 PMC3328488

[psyp70144-bib-0006] Bishop, V. S. , A. Malliani , and P. Thorén . 1983. “Cardiac Mechanoreceptors.” In Handbook of Physiology, edited by J. T. Shephard and F. M. Abbound , 497–555. Waverly Press. 10.1002/cphy.cp020315.

[psyp70144-bib-0007] Brainard, D. H. 1997. “The Psychophysics Toolbox.” Spatial Vision 10: 433–436. 9176952

[psyp70144-bib-0008] Bullock, T. , H. Cecotti , and B. Giesbrecht . 2015. “Multiple Stages of Information Processing Are Modulated During Acute Bouts of Exercise.” Neuroscience 307: 138–150. 26318337 10.1016/j.neuroscience.2015.08.046

[psyp70144-bib-0009] Bullock, T. , J. C. Elliott , J. T. Serences , and B. Giesbrecht . 2017. “Acute Exercise Modulates Feature‐Selective Responses in Human Cortex.” Journal of Cognitive Neuroscience 29: 605–618. 27897672 10.1162/jocn_a_01082

[psyp70144-bib-0010] Cantelon, J. A. , and G. E. Giles . 2021. “A Review of Cognitive Changes During Acute Aerobic Exercise.” Frontiers in Psychology 12: 653158. 34975602 10.3389/fpsyg.2021.653158 PMC8716584

[psyp70144-bib-0011] Cao, L. , and B. Händel . 2019. “Walking Enhances Peripheral Visual Processing in Humans.” PLoS Biology 17: e3000511. 31603894 10.1371/journal.pbio.3000511 PMC6808500

[psyp70144-bib-0012] Chang, Y. K. , J. D. Labban , J. I. Gapin , and J. L. Etnier . 2012. “The Effects of Acute Exercise on Cognitive Performance: A Meta‐Analysis.” Brain Research 1453: 87–101. 22480735 10.1016/j.brainres.2012.02.068

[psyp70144-bib-0013] Chen, X. , L. Cao , and B. F. Haendel . 2022. “Human Visual Processing During Walking: Dissociable Pre‐ and Post‐Stimulus Influences.” NeuroImage 264: 119757. 36414209 10.1016/j.neuroimage.2022.119757 PMC9771827

[psyp70144-bib-0014] Ciria, L. F. , A. Luque‐Casado , D. Sanabria , D. Holgado , P. C. Ivanov , and P. Perakakis . 2019. “Oscillatory Brain Activity During Acute Exercise: Tonic and Transient Neural Response to an Oddball Task.” Psychophysiology 56: e13326. 30637763 10.1111/psyp.13326 PMC7311047

[psyp70144-bib-0015] Clark, J. H. 1924. “The Ishihara Test for Color Blindness.” American Journal of Physiological Optics 5: 269–276.

[psyp70144-bib-0016] Cobos, M. I. , P. M. Guerra , J. Vila , and A. B. Chica . 2019. “Heart‐Rate Modulations Reveal Attention and Consciousness Interactions.” Psychophysiology 56: e13295. 30362275 10.1111/psyp.13295

[psyp70144-bib-0017] Coll, M.‐P. , H. Hobson , G. Bird , and J. Murphy . 2021. “Systematic Review and Meta‐Analysis of the Relationship Between the Heartbeat‐Evoked Potential and Interoception.” Neuroscience & Biobehavioral Reviews 122: 190–200. 33450331 10.1016/j.neubiorev.2020.12.012

[psyp70144-bib-0018] Corcoran, A. W. , V. G. Macefield , and J. Hohwy . 2021. “Be Still My Heart: Cardiac Regulation as a Mode of Uncertainty Reduction.” Psychonomic Bulletin & Review 28: 1211–1223. 33755894 10.3758/s13423-021-01888-y

[psyp70144-bib-0019] Crabbe, J. B. , and R. K. Dishman . 2004. “Brain Electrocortical Activity During and After Exercise: A Quantitative Synthesis.” Psychophysiology 41: 563–574. 15189479 10.1111/j.1469-8986.2004.00176.x

[psyp70144-bib-0020] Dampney, R. A. L. , J. W. Polson , P. D. Potts , Y. Hirooka , and J. Horiuchi . 2003. “Functional Organization of Brain Pathways Subserving the Baroreceptor Reflex: Studies in Conscious Animals Using Immediate Early Gene Expression.” Cellular and Molecular Neurobiology 23: 597–616. 14514018 10.1023/A:1025080314925 PMC11530187

[psyp70144-bib-0021] De Sanctis, P. , J. S. Butler , B. R. Malcolm , and J. J. Foxe . 2014. “Recalibration of Inhibitory Control Systems During Walking‐Related Dual‐Task Interference: A Mobile Brain‐Body Imaging (MOBI) Study.” NeuroImage 94: 55–64. 24642283 10.1016/j.neuroimage.2014.03.016 PMC4209901

[psyp70144-bib-0022] Enders, H. , F. Cortese , C. Maurer , J. Baltich , A. B. Protzner , and B. M. Nigg . 2016. “Changes in Cortical Activity Measured With EEG During a High‐Intensity Cycling Exercise.” Journal of Neurophysiology 115: 379–388. 26538604 10.1152/jn.00497.2015 PMC4760484

[psyp70144-bib-0023] Ferris, L. T. , J. S. Williams , and C.‐L. Shen . 2007. “The Effect of Acute Exercise on Serum Brain‐Derived Neurotrophic Factor Levels and Cognitive Function.” Medicine and Science in Sports and Exercise 39: 728–734. 17414812 10.1249/mss.0b013e31802f04c7

[psyp70144-bib-0024] Ford, T. W. , and P. A. Kirkwood . 2018. “Cardiac Modulation of Alpha Motoneuron Discharges.” Journal of Neurophysiology 119: 1723–1730. 29412777 10.1152/jn.00025.2018 PMC6008091

[psyp70144-bib-0025] Foxe, J. J. , and A. C. Snyder . 2011. “The Role of Alpha‐Band Brain Oscillations as a Sensory Suppression Mechanism During Selective Attention.” Frontiers in Psychology 2: 154. 21779269 10.3389/fpsyg.2011.00154 PMC3132683

[psyp70144-bib-0026] Fumoto, M. , T. Oshima , K. Kamiya , et al. 2010. “Ventral Prefrontal Cortex and Serotonergic System Activation During Pedaling Exercise Induces Negative Mood Improvement and Increased Alpha Band in EEG.” Behavioural Brain Research 213: 1–9. 20412817 10.1016/j.bbr.2010.04.017

[psyp70144-bib-0027] Garber, C. E. , B. Blissmer , M. R. Deschenes , et al. 2011. “Quantity and Quality of Exercise for Developing and Maintaining Cardiorespiratory, Musculoskeletal, and Neuromotor Fitness in Apparently Healthy Adults: Guidance for Prescribing Exercise.” Medicine and Science in Sports and Exercise 43: 1334–1359. 21694556 10.1249/MSS.0b013e318213fefb

[psyp70144-bib-0028] Garrett, J. , C. Chak , T. Bullock , and B. Giesbrecht . 2024. “A Systematic Review and Bayesian Meta‐Analysis Provide Evidence for an Effect of Acute Physical Activity on Cognition in Young Adults.” Communications Psychology 2: 82. 39242965 10.1038/s44271-024-00124-2 PMC11358546

[psyp70144-bib-0029] Gray, M. A. , P. Taggart , P. M. Sutton , et al. 2007. “A Cortical Potential Reflecting Cardiac Function.” Proceedings of the National Academy of Sciences 104: 6818–6823. 10.1073/pnas.0609509104 PMC1871868 17420478

[psyp70144-bib-0030] Grund, M. , E. al , M. Pabst , et al. 2022. “Respiration, Heartbeat, and Conscious Tactile Perception.” Journal of Neuroscience 42: 643–656. 34853084 10.1523/JNEUROSCI.0592-21.2021 PMC8805629

[psyp70144-bib-0031] Hatfield, B. D. , T. W. Spalding , D. L. Santa Maria , et al. 1998. “Respiratory Sinus Arrhythmia During Exercise in Aerobically Trained and Untrained Men.” Medicine and Science in Sports and Exercise 30: 206–214. 9502347 10.1097/00005768-199802000-00006

[psyp70144-bib-0032] Heinonen, I. , K. K. Kalliokoski , J. C. Hannukainen , D. J. Duncker , P. Nuutila , and J. Knuuti . 2014. “Organ‐Specific Physiological Responses to Acute Physical Exercise and Long‐Term Training in Humans.” Physiology 29: 421–436. 25362636 10.1152/physiol.00067.2013

[psyp70144-bib-0033] Hosang, L. , E. Mouchlianitis , S. M. R. Guérin , and C. I. Karageorghis . 2022. “Effects of Exercise on Electroencephalography‐Recorded Neural Oscillations: A Systematic Review.” International Review of Sport and Exercise Psychology 0: 1–54.

[psyp70144-bib-0034] Hottenrott, K. , M. Taubert , and T. Gronwald . 2013. “Cortical Brain Activity Is Influenced by Cadence in Cyclists.” Open Sports Sciences Journal 6: 9–14. 10.1080/02640414.2016.1198045 27328649

[psyp70144-bib-0035] Iemi, L. , M. Chaumon , S. M. Crouzet , and N. A. Busch . 2017. “Spontaneous Neural Oscillations Bias Perception by Modulating Baseline Excitability.” Journal of Neuroscience 37: 807–819. 28123017 10.1523/JNEUROSCI.1432-16.2016 PMC6597018

[psyp70144-bib-0036] Jammal Salameh, L. , S. H. Bitzenhofer , I. L. Hanganu‐Opatz , M. Dutschmann , and V. Egger . 2024. “Blood Pressure Pulsations Modulate Central Neuronal Activity via Mechanosensitive Ion Channels.” Science 383: eadk8511. 38301001 10.1126/science.adk8511

[psyp70144-bib-0037] Jänig, W. 1996. “Neurobiology of Visceral Afferent Neurons: Neuroanatomy, Functions, Organ Regulations and Sensations.” Biological Psychology 42: 29–51. 8770369 10.1016/0301-0511(95)05145-7

[psyp70144-bib-0038] Jennings, J. R. , and C. C. Wood . 1977. “Cardiac Cycle Time Effects on Performance, Phasic Cardiac Responses, and Their Intercorrelation in Choice Reaction Time.” Psychophysiology 14: 297–307. 854559 10.1111/j.1469-8986.1977.tb01179.x

[psyp70144-bib-0039] Jensen, O. , and A. Mazaheri . 2010. “Shaping Functional Architecture by Oscillatory Alpha Activity: Gating by Inhibition.” Frontiers in Human Neuroscience 4: 186. 21119777 10.3389/fnhum.2010.00186 PMC2990626

[psyp70144-bib-0040] Kefauver, J. M. , A. B. Ward , and A. Patapoutian . 2020. “Discoveries in Structure and Physiology of Mechanically Activated Ion Channels.” Nature 587: 567–576. 33239794 10.1038/s41586-020-2933-1 PMC8477435

[psyp70144-bib-0041] Kelly, S. P. , E. C. Lalor , R. B. Reilly , and J. J. Foxe . 2006. “Increases in Alpha Oscillatory Power Reflect an Active Retinotopic Mechanism for Distracter Suppression During Sustained Visuospatial Attention.” Journal of Neurophysiology 95: 3844–3851. 16571739 10.1152/jn.01234.2005

[psyp70144-bib-0042] Kern, M. , A. Aertsen , A. Schulze‐Bonhage , and T. Ball . 2013. “Heart Cycle‐Related Effects on Event‐Related Potentials, Spectral Power Changes, and Connectivity Patterns in the Human Ecog.” NeuroImage 81: 178–190. 23684883 10.1016/j.neuroimage.2013.05.042

[psyp70144-bib-0043] Kim, K. J. , J. Ramiro Diaz , J. A. Iddings , and J. A. Filosa . 2016. “Vasculo‐Neuronal Coupling: Retrograde Vascular Communication to Brain Neurons.” Journal of Neuroscience 36: 12624–12639. 27821575 10.1523/JNEUROSCI.1300-16.2016 PMC5157107

[psyp70144-bib-0044] Kleiner, M. , D. Brainard , and D. Pelli . 2007. “What's New in Psychtoolbox‐3.” Perception 36: 1–16.

[psyp70144-bib-0045] Klimesch, W. , P. Sauseng , and S. Hanslmayr . 2007. “EEG Alpha Oscillations: The Inhibition–Timing Hypothesis.” Brain Research Reviews 53: 63–88. 16887192 10.1016/j.brainresrev.2006.06.003

[psyp70144-bib-0046] Kluger, D. S. , E. Balestrieri , N. A. Busch , and J. Gross . 2021. “Respiration Aligns Perception With Neural Excitability.” eLife 10: e70907. 34904567 10.7554/eLife.70907 PMC8763394

[psyp70144-bib-0047] Koriath, J. J. , E. Lindholm , and D. M. Landers . 1987. “Cardiac‐Related Cortical Activity During Variations in Mean Heart Rate.” International Journal of Psychophysiology 5: 289–299. 3436846 10.1016/0167-8760(87)90060-2

[psyp70144-bib-0048] Lacey, B. C. , and J. I. Lacey . 1974. “Studies of Heart Rate and Other Bodily Processes in Sensorimotor Behavior.” In Cardiovascular Psychophysiology: Current Issues in Response Mechanisms, Biofeedback and Methodology, 538–564. AldineTransaction.

[psyp70144-bib-0049] Lacey, B. C. , and J. I. Lacey . 1978. “Two‐Way Communication Between the Heart and the Brain: Significance of Time Within the Cardiac Cycle.” American Psychologist 33: 99–113. 637402 10.1037//0003-066x.33.2.99

[psyp70144-bib-0050] Lacey, B. C. , and J. I. Lacey . 1980. “Cognitive Modulation of Time‐Dependent Primary Bradycardia.” Psychophysiology 17: 209–221. 7384370 10.1111/j.1469-8986.1980.tb00137.x

[psyp70144-bib-0051] Lacey, J. I. 1967. “Somatic Response Patterning and Stress: Some Revisions of Activation Theory.” In Pyschological Stress: Issues in Research, edited by M. H. Appley and R. Trimbull . Appleton Century‐Crofts.

[psyp70144-bib-0052] Lacey, J. I. , and B. C. Lacey . 1958. “The Relationship of Resting Autonomic Activity to Motor Impulsivity.” Research Publications ‐ Association for Research in Nervous and Mental Disease 36: 144–209. 13527784

[psyp70144-bib-0053] Lacey, J. I. , and B. C. Lacey . 1970. “Some Autonomic‐Central Nervous System Interrelationships.” In Physiological Correlates of Emotion, edited by P. Black , 205–227. Academic Press. 10.1016/B978-0-12-102850-3.50016-5.

[psyp70144-bib-0054] Lambourne, K. , and P. Tomporowski . 2010. “The Effect of Exercise‐Induced Arousal on Cognitive Task Performance: A Meta‐Regression Analysis.” Brain Research 1341: 12–24. 20381468 10.1016/j.brainres.2010.03.091

[psyp70144-bib-0055] Leutheuser, H. , S. Gradl , L. Anneken , et al. 2016. Instantaneous P‐ and T‐Wave Detection: Assessment of Three ECG Fiducial Points Detection Algorithms. IEEE Xplore. 10.1109/BSN.2016.7516283.

[psyp70144-bib-0056] Macefield, V. G. 2003. “Cardiovascular and Respiratory Modulation of Tactile Afferents in the Human Finger Pad.” Experimental Physiology 88: 617–625. 12955162 10.1113/eph8802548

[psyp70144-bib-0057] Marina, N. , I. N. Christie , A. Korsak , et al. 2020. “Astrocytes Monitor Cerebral Perfusion and Control Systemic Circulation to Maintain Brain Blood Flow.” Nature Communications 11: 131. 10.1038/s41467-019-13956-y PMC6952443 31919423

[psyp70144-bib-0058] Maris, E. , and R. Oostenveld . 2007. “Nonparametric Statistical Testing of EEG‐ and MEG‐Data.” Journal of Neuroscience Methods 164: 177–190. 17517438 10.1016/j.jneumeth.2007.03.024

[psyp70144-bib-0059] Meeusen, R. , and K. De Meirleir . 1995. “Exercise and Brain Neurotransmission.” Sports Medicine 20: 160–188. 8571000 10.2165/00007256-199520030-00004

[psyp70144-bib-0060] Mehling, W. E. , C. Price , J. J. Daubenmier , M. Acree , E. Bartmess , and A. Stewart . 2012. “The Multidimensional Assessment of Interoceptive Awareness (MAIA).” PLoS One 7: e48230. 23133619 10.1371/journal.pone.0048230 PMC3486814

[psyp70144-bib-0061] Mongin, D. , C. Chabert , M. G. Extremera , et al. 2022. “Decrease of Heart Rate Variability During Exercise: An Index of Cardiorespiratory Fitness.” PLoS One 17: e0273981. 36054204 10.1371/journal.pone.0273981 PMC9439241

[psyp70144-bib-0062] Montoya, P. , R. Schandry , and A. Müller . 1993. “Heartbeat Evoked Potentials (HEP): Topography and Influence of Cardiac Awareness and Focus of Attention.” Electroencephalography and Clinical Neurophysiology/Evoked Potentials Section 88: 163–172. 10.1016/0168-5597(93)90001-6 7684965

[psyp70144-bib-0063] Motyka, P. , M. Grund , N. Forschack , E. al , A. Villringer , and M. Gaebler . 2019. “Interactions Between Cardiac Activity and Conscious Somatosensory Perception.” Psychophysiology 56: e13424. 31245848 10.1111/psyp.13424

[psyp70144-bib-0064] Nes, B. M. , I. Janszky , U. Wisløff , A. Støylen , and T. Karlsen . 2013. “Age‐Predicted Maximal Heart Rate in Healthy Subjects: The HUNT Fitness Study.” Scandinavian Journal of Medicine & Science in Sports 23: 697–704. 22376273 10.1111/j.1600-0838.2012.01445.x

[psyp70144-bib-0065] Noble, D. J. , and S. Hochman . 2019. “Hypothesis: Pulmonary Afferent Activity Patterns During Slow, Deep Breathing Contribute to the Neural Induction of Physiological Relaxation.” Frontiers in Physiology 10: 1176. 31572221 10.3389/fphys.2019.01176 PMC6753868

[psyp70144-bib-0066] Oldfield, R. C. 1971. “The Assessment and Analysis of Handedness: The Edinburgh Inventory.” Neuropsychologia 9: 97–113. 5146491 10.1016/0028-3932(71)90067-4

[psyp70144-bib-0067] Oostenveld, R. , P. Fries , E. Maris , and J.‐M. Schoffelen . 2011. “FieldTrip: Open Source Software for Advanced Analysis of MEG, EEG, and Invasive Electrophysiological Data.” Computational Intelligence and Neuroscience 2011: 1–9. 21253357 10.1155/2011/156869 PMC3021840

[psyp70144-bib-0068] Pan, J. , and W. J. Tompkins . 1985. “A Real‐Time QRS Detection Algorithm.” IEEE Transactions on Biomedical Engineering 32: 230–236. 3997178 10.1109/TBME.1985.325532

[psyp70144-bib-0069] Park, H.‐D. , and O. Blanke . 2019. “Heartbeat‐Evoked Cortical Responses: Underlying Mechanisms, Functional Roles, and Methodological Considerations.” NeuroImage 197: 502–511. 31051293 10.1016/j.neuroimage.2019.04.081

[psyp70144-bib-0070] Park, H.‐D. , S. Correia , A. Ducorps , and C. Tallon‐Baudry . 2014. “Spontaneous Fluctuations in Neural Responses to Heartbeats Predict Visual Detection.” Nature Neuroscience 17: 612–618. 24609466 10.1038/nn.3671

[psyp70144-bib-0071] Pennebaker, J. W. , and J. M. Lightner . 1980. “Competition of Internal and External Information in an Exercise Setting.” Journal of Personality and Social Psychology 39: 165–174. 7411392 10.1037//0022-3514.39.1.165

[psyp70144-bib-0072] Perogamvros, L. , H. D. Park , L. Bayer , A. A. Perrault , O. Blanke , and S. Schwartz . 2019. “Increased Heartbeat‐Evoked Potential During REM Sleep in Nightmare Disorder.” NeuroImage: Clinical 22: 101701. 30739843 10.1016/j.nicl.2019.101701 PMC6370851

[psyp70144-bib-0073] Petzschner, F. H. , L. A. Weber , K. V. Wellstein , G. Paolini , C. T. do , and K. E. Stephan . 2019. “Focus of Attention Modulates the Heartbeat Evoked Potential.” NeuroImage 186: 595–606. 30472370 10.1016/j.neuroimage.2018.11.037

[psyp70144-bib-0074] Poland, E. , A. Bhonsle , I. Steinmann , and M. Wilke . 2021. “Reduced Alpha Amplitudes Predict Perceptual Suppression.” Scientific Reports 11: 13040. 34158567 10.1038/s41598-021-92404-8 PMC8219776

[psyp70144-bib-0075] Pollatos, O. , W. Kirsch , and R. Schandry . 2005. “Brain Structures Involved in Interoceptive Awareness and Cardioafferent Signal Processing: A Dipole Source Localization Study.” Human Brain Mapping 26: 54–64. 15852466 10.1002/hbm.20121 PMC6871699

[psyp70144-bib-0076] Pollatos, O. , and R. Schandry . 2004. “Accuracy of Heartbeat Perception Is Reflected in the Amplitude of the Heartbeat‐Evoked Brain Potential.” Psychophysiology 41: 476–482. 15102134 10.1111/1469-8986.2004.00170.x

[psyp70144-bib-0077] Querido, J. S. , and A. W. Sheel . 2007. “Regulation of Cerebral Blood Flow During Exercise.” Sports Medicine 37: 765–782. 17722948 10.2165/00007256-200737090-00002

[psyp70144-bib-0078] Romei, V. , V. Brodbeck , C. Michel , A. Amedi , A. Pascual‐Leone , and G. Thut . 2008. “Spontaneous Fluctuations in Posterior Alpha‐Band EEG Activity Reflect Variability in Excitability of Human Visual Areas.” Cerebral Cortex 18: 2010–2018. 18093905 10.1093/cercor/bhm229 PMC2517102

[psyp70144-bib-0079] Romei, V. , T. Rihs , V. Brodbeck , and G. Thut . 2008. “Resting Electroencephalogram Alpha‐Power Over Posterior Sites Indexes Baseline Visual Cortex Excitability.” Neuroreport 19: 203–208. 18185109 10.1097/WNR.0b013e3282f454c4

[psyp70144-bib-0080] Sandman, C. A. , T. R. McCanne , D. N. Kaiser , and B. Diamond . 1977. “Heart Rate and Cardiac Phase Influences on Visual Perception.” Journal of Comparative and Physiological Psychology 91: 189–202. 838914 10.1037/h0077302

[psyp70144-bib-0081] Sauseng, P. , W. Klimesch , W. Stadler , et al. 2005. “A Shift of Visual Spatial Attention Is Selectively Associated With Human EEG Alpha Activity.” European Journal of Neuroscience 22: 2917–2926. 16324126 10.1111/j.1460-9568.2005.04482.x

[psyp70144-bib-0082] Schandry, R. , and P. Montoya . 1996. “Event‐Related Brain Potentials and the Processing of Cardiac Activity.” Biological Psychology 42: 75–85. 8770371 10.1016/0301-0511(95)05147-3

[psyp70144-bib-0083] Schandry, R. , B. Sparrer , and R. Weitkunat . 1986. “From the Heart to the Brain: A Study of Heartbeat Contingent Scalp Potentials.” International Journal of Neuroscience 30: 261–275. 3793380 10.3109/00207458608985677

[psyp70144-bib-0084] Sedghamiz, H. 2014. “Matlab Implementation of Pan Tompkins ECG QRS Detector.” 10.13140/RG.2.2.14202.59841.

[psyp70144-bib-0085] Skora, L. I. , J. J. A. Livermore , and K. Roelofs . 2022. “The Functional Role of Cardiac Activity in Perception and Action.” Neuroscience & Biobehavioral Reviews 137: 104655. 35395334 10.1016/j.neubiorev.2022.104655

[psyp70144-bib-0086] Somsen, R. J. M. , J. R. Jennings , and M. W. Van der Molen . 2004. “The Cardiac Cycle Time Effect Revisited: Temporal Dynamics of the Central‐Vagal Modulation of Heart Rate in Human Reaction Time Tasks.” Psychophysiology 41: 941–953. 15563347 10.1111/j.1469-8986.2004.00241.x

[psyp70144-bib-0087] Storzer, L. , M. Butz , J. Hirschmann , et al. 2016. “Bicycling and Walking Are Associated With Different Cortical Oscillatory Dynamics.” Frontiers in Human Neuroscience 10: 61. 26924977 10.3389/fnhum.2016.00061 PMC4759288

[psyp70144-bib-0088] Thut, G. , A. Nietzel , S. A. Brandt , and A. Pascual‐Leone . 2006. “Alpha‐Band Electroencephalographic Activity Over Occipital Cortex Indexes Visuospatial Attention Bias and Predicts Visual Target Detection.” Journal of Neuroscience 26: 9494–9502. 16971533 10.1523/JNEUROSCI.0875-06.2006 PMC6674607

[psyp70144-bib-0089] Vázquez‐Seisdedos, C. R. , J. E. Neto , E. J. Marañón Reyes , A. Klautau , and R. C. Limão de Oliveira . 2011. “New Approach for T‐Wave End Detection on Electrocardiogram: Performance in Noisy Conditions.” Biomedical Engineering Online 10: 77. 21906317 10.1186/1475-925X-10-77 PMC3201026

[psyp70144-bib-0090] Vila, J. , P. Guerra , M. Á. Muñoz , et al. 2007. “Cardiac Defense: From Attention to Action.” International Journal of Psychophysiology 66: 169–182. 17706311 10.1016/j.ijpsycho.2007.07.004

[psyp70144-bib-0091] Villena‐González, M. , C. Moënne‐Loccoz , R. A. Lagos , et al. 2017. “Attending to the Heart Is Associated With Posterior Alpha Band Increase and a Reduction in Sensitivity to Concurrent Visual Stimuli.” Psychophysiology 54: 1483–1497. 28560781 10.1111/psyp.12894

[psyp70144-bib-0092] Wagner, J. , T. Solis‐Escalante , R. Scherer , C. Neuper , and G. Müller‐Putz . 2014. “It's How You Get There: Walking Down a Virtual Alley Activates Premotor and Parietal Areas.” Frontiers in Human Neuroscience 8: 93. 24611043 10.3389/fnhum.2014.00093 PMC3933811

[psyp70144-bib-0093] Wallman‐Jones, A. , P. Perakakis , M. Tsakiris , and M. Schmidt . 2021. “Physical Activity and Interoceptive Processing: Theoretical Considerations for Future Research.” International Journal of Psychophysiology 166: 38–49. 33965423 10.1016/j.ijpsycho.2021.05.002

[psyp70144-bib-0094] Wang, J. , and O. P. Hamill . 2021. “Piezo2‐Peripheral Baroreceptor Channel Expressed in Select Neurons of the Mouse Brain: A Putative Mechanism for Synchronizing Neural Networks by Transducing Intracranial Pressure Pulses.” Journal of Integrative Neuroscience 20: 825–837. 34997707 10.31083/j.jin2004085

[psyp70144-bib-0095] Wilke, M. , N. K. Logothetis , and D. A. Leopold . 2003. “Generalized Flash Suppression of Salient Visual Targets.” Neuron 39: 1043–1052. 12971902 10.1016/j.neuron.2003.08.003

[psyp70144-bib-0096] Worden, M. S. , J. J. Foxe , N. Wang , and G. V. Simpson . 2000. “Anticipatory Biasing of Visuospatial Attention Indexed by Retinotopically Specific Alpha‐Band Electroencephalography Increases Over Occipital Cortex.” Journal of Neuroscience 20: RC63. 10704517 10.1523/JNEUROSCI.20-06-j0002.2000 PMC6772495

[psyp70144-bib-0097] Wöstmann, M. , M. Alavash , and J. Obleser . 2019. “Alpha Oscillations in the Human Brain Implement Distractor Suppression Independent of Target Selection.” Journal of Neuroscience 39: 9797–9805. 31641052 10.1523/JNEUROSCI.1954-19.2019 PMC6891068

[psyp70144-bib-0098] Zeng, W.‐Z. , K. L. Marshall , S. Min , et al. 2018. “PIEZOs Mediate Neuronal Sensing of Blood Pressure and the Baroreceptor Reflex.” Science 362: 464–467. 30361375 10.1126/science.aau6324 PMC6563913

