# Supplementary material for: Neural and Cardiac Contributions to Perceptual Suppression During Cycling
Source: Psychophysiology. 2025 Sep 9;62(9):e70144. doi: 10.1111/psyp.70144 (PMC12421089; doi:10.1111/psyp.70144)
Supplement: Supplementary file 1 — Data S1: Supporting Information. [file PSYP-62-e70144-s001.docx]

# Supplementary Information

| 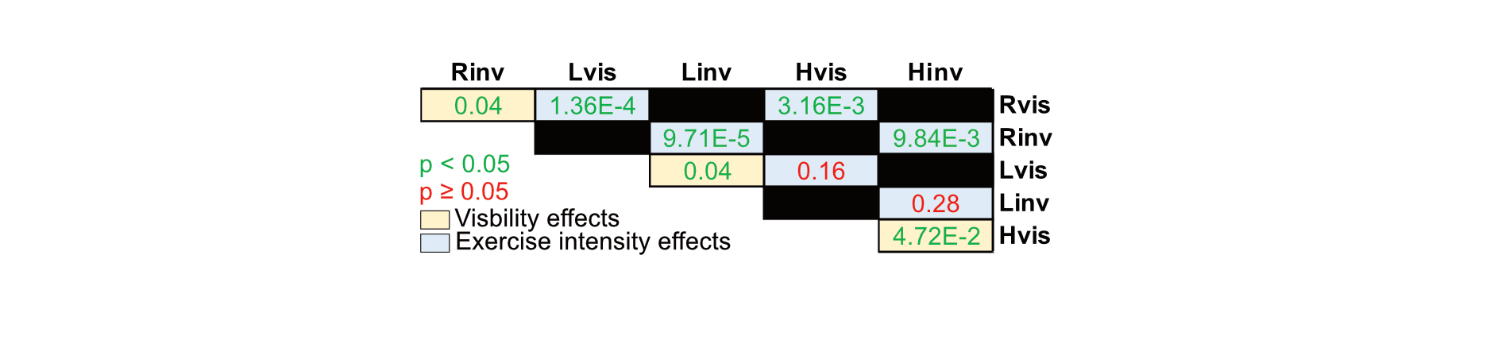  Table S1 - Table of uncorrected *p* values for pairwise comparison of pre-RDM parieto-occipital alpha amplitudes across exercise intensity and visibility conditions |
| --- |
| These uncorrected *p* values correspond to the comparisons reported in **Figure 6**, testing for the effects of target visibility (visible – vis; invisible - inv) and exercise intensity (rest – R; low-resistance – L, high-resistance - H) on pre-RDM parieto-occipital alpha amplitudes using Wilcoxon signed-rank tests. These results indicate that alpha amplitudes reflect target visibility and that exercise influences alpha amplitudes compared to rest, although no significant differences were observed between the two cycling conditions. |


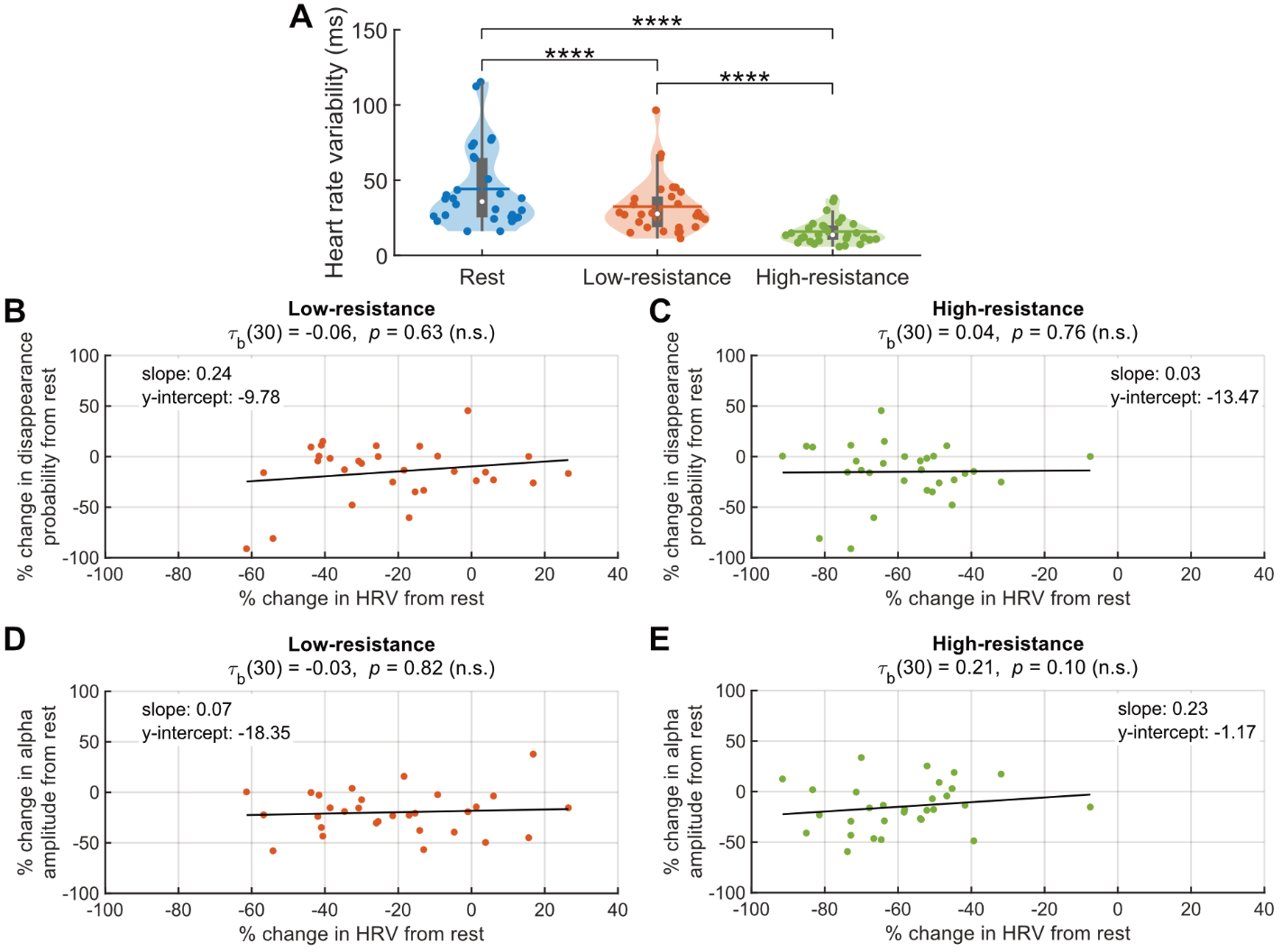


Figure S1 – RMSSD heart rate variability (HRV) decreases with cycling intensity

(**A**) Violin plots showing the pre-RDM RMSSD HRV during GFS performed at rest (blue), low-resistance (orange), and high-resistance (green) cycling. Vertical grey boxes indicate the interquartile range (IQR), with grey whiskers indicating 1.5 $\times$ IQR, surrounded on each side by the kernel density estimation in the colour corresponding to each group. Horizontal lines, in the colour corresponding to each group, denote means, and white dots indicate medians. Each dot in a given condition represents the average value of a single subject. Statistical significance between conditions, as assessed by Wilcoxon signed-rank tests with a Bonferroni adjustment, is indicated at the *p* $\leq$ 0.0001 (****) level. Correlations as assessed by Kendall’s Tau-b between percent change in HRV from rest and percent change in disappearance probability from rest for (**B**) low-resistance and (**C**) high-resistance cycling. Correlations between percent change in HRV from rest and percent change in alpha amplitude from rest for (**D**) low-resistance and (**E**) high-resistance cycling. All correlations are not significant. *N* = 30 for all plots.

| 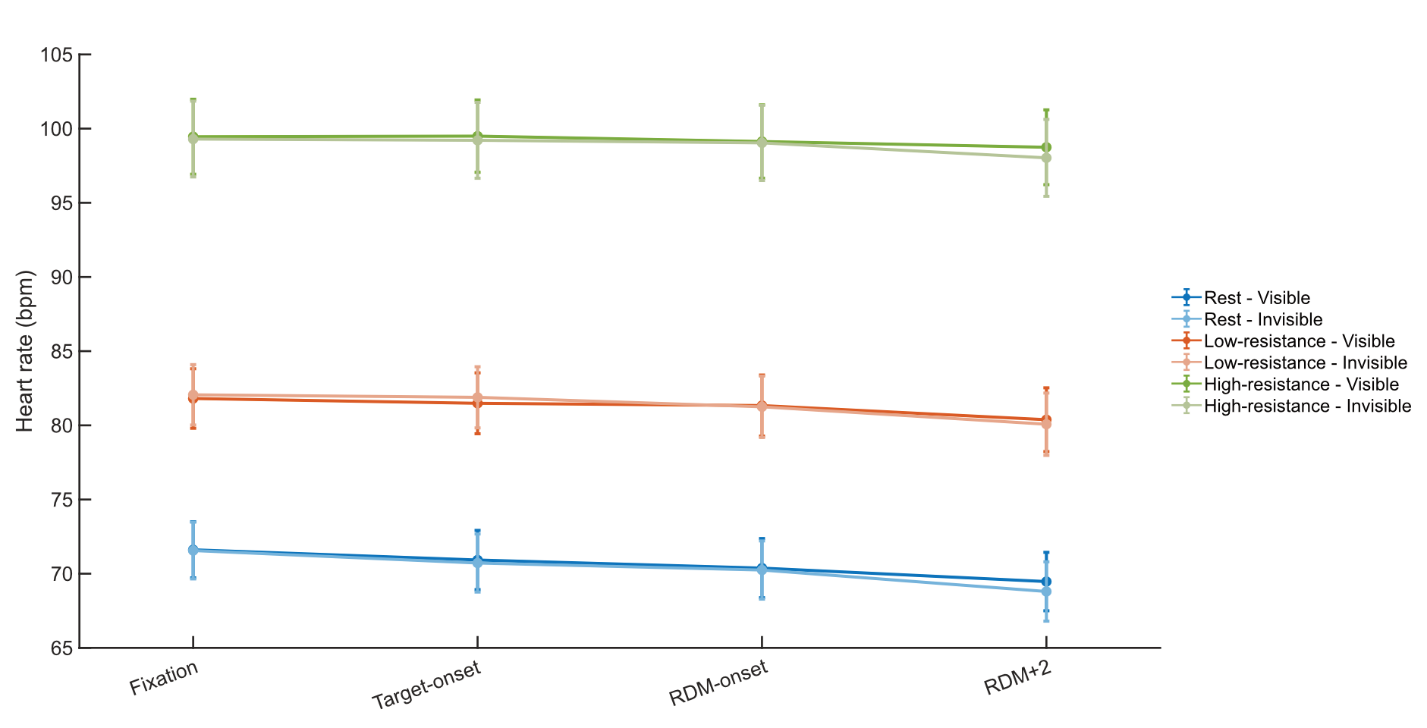  Figure S2 – Phasic heart rate deceleration over the course of the GFS trial |
| --- |
| Phasic heart rate changes across the course of a trial were assessed using a three-way repeated measures ANOVA with exercise intensity (levels: rest, low-resistance cycling, high-resistance cycling), target visibility (levels: visible, invisible), and time (levels: fixation onset, target onset, RDM onset, trial end (RDM + 2s)) as factors. The analysis showed significant main effects of exercise intensity (Greenhouse-Geisser corrected; *F*(1.25, 36.38) = 269.60, *p* = 9.28$\times$10^-20^, $\eta_{p}^{2}$ = 0.90) and time (Greenhouse-Geisser corrected; *F*(1.68, 48.64) = 22.65, *p* = 4.83$\times$10^-7^, $\eta_{p}^{2}$ = 0.49), but not target visibility (*F*(1, 29) = 1.26, *p* = 0.27, $\eta_{p}^{2}$ = 0.04). There were significant two-way interactions between exercise intensity and time (Greenhouse-Geisser corrected; *F*(3.56, 103.30) = 4.30, *p* = 4.20$\times$10^-3^, $\eta_{p}^{2}$ = 0.13) and target visibility and time (Greenhouse-Geisser corrected; *F*(2.60, 75.25) = 5.74, *p* = 2.23$\times$10^-3^, $\eta_{p}^{2}$ = 0.17), but not between exercise intensity and target visibility (*F*(2, 58) = 0.92, *p* = 0.40, $\eta_{p}^{2}$ = 0.03). There was also no significant three-way interaction between the factors (*F*(6, 174) = 0.57, *p* = 0.76, $\eta_{p}^{2}$ = 0.02).  The table of the Bonferroni-corrected pairwise comparisons for the factor of time is presented below. These results indicate that there was a decrease in heart rate from fixation to target onset, indicative of the well-characterised phenomenon of cardiac deceleration in anticipation of an upcoming stimulus or response registration, with fixation serving as a ‘warning’ of an upcoming stimulus, i.e. the onset of a target, which in turn requires a response. Similarly, there is also a slight, non-significant decrease in heart rate from target onset to RDM onset. It is likely that the cardiac acceleration typically reported after response registration does not occur in this case since the target onset acts as a warning of the upcoming RDM onset. Furthermore, there is a potential for an additional response registration after RDM onset if the target is perceptually suppressed. However, since subjects were instructed to also report if the target reappeared, the cardiac acceleration typically reported after response registration, is not observed; rather, it appears that the heart rate continues to decrease after RDM onset until the end of the trial. |

Table S2 - Bonferroni-corrected pairwise comparisons for the factor of time

| Factor: Time | | | | *p* | 95% Confidence Interval for difference | |
| --- | --- | --- | --- | --- | --- | --- |
| Level (I) | Level (J) | Mean difference in heart rate (I-J) | Std. Error |  | Lower bound | Upper bound |
| Fixation | Target onset | 0.34 | 0.11 | 0.03 | 0.02 | 0.67 |
|  | RDM onset | 0.76 | 0.27 | 0.06 | -0.03 | 1.50 |
|  | End of trial | 1.72 | 0.26 | 2.24$\times$10^-6^ | 0.97 | 2.46 |
| Target onset | RDM onset | 0.39 | 0.22 | 5.02$\times$10^-2^ | -0.23 | 1.01 |
|  | End of trial | 1.37 | 0.25 | 4.31$\times$10^-5^ | 0.66 | 2.08 |
| RDM onset | End of trial | 0.98 | 0.16 | 8.31$\times$10^-6^ | 0.52 | 1.44 |

| 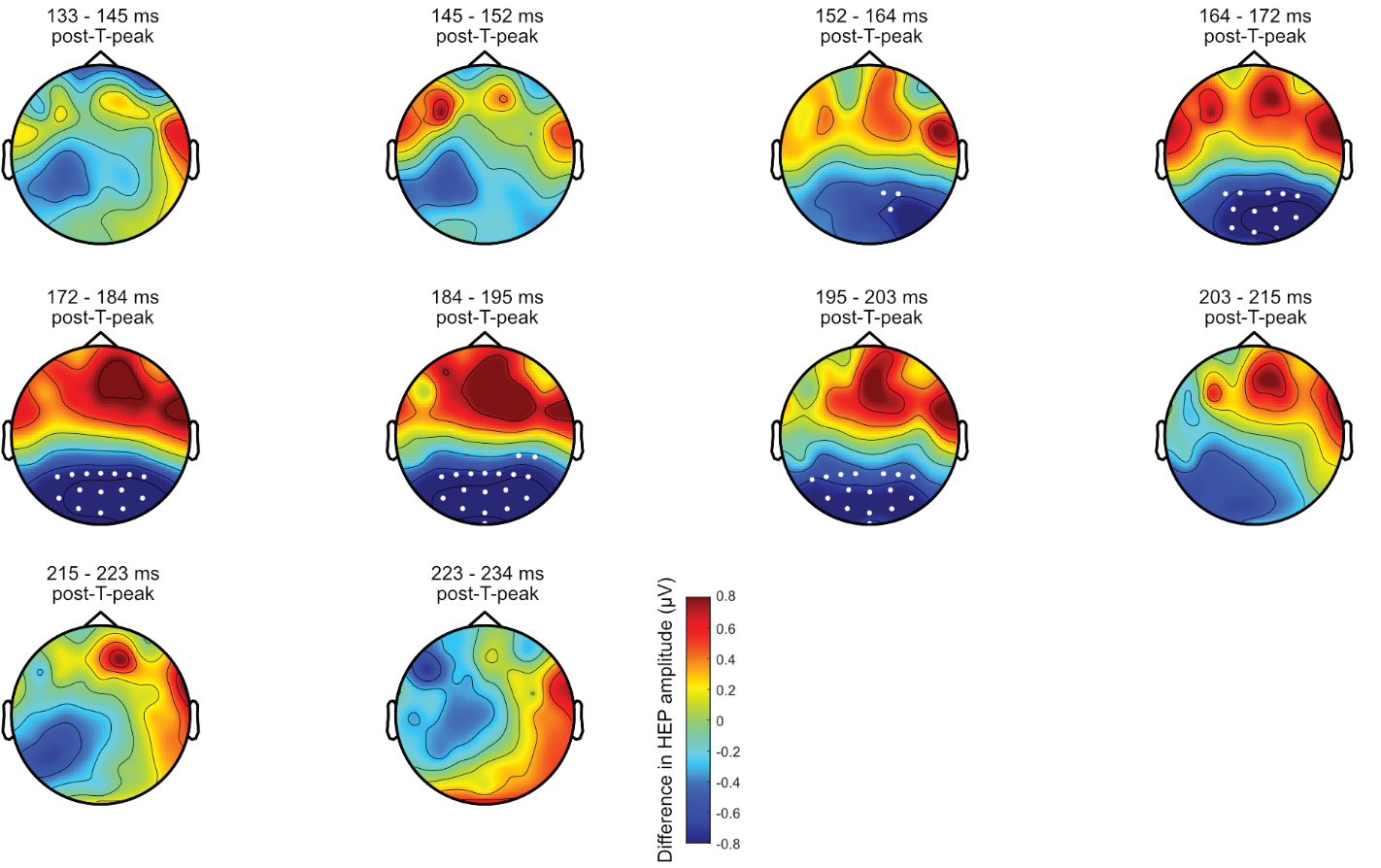Scalp topographies of the low-resistance – rest difference in HEP amplitude for 12 ms windows spanning the 133 ms to 234 ms post-T-peak time window. The T-peaks chosen for this analysis were at least 300 ms after target onset and up to 400 ms before RDM onset, to avoid the HEPs from being contaminated by responses to the target or the RDM onset. Spatio-temporal clusters showing a significant difference between the two exercise intensity conditions are indicated by white dots (*p* < 0.05).  Figure S3 – Cluster based permutation analysis of Heartbeat Evoked Potentials (HEP) investigating effect of exercise on HEP amplitude |
| --- |

| 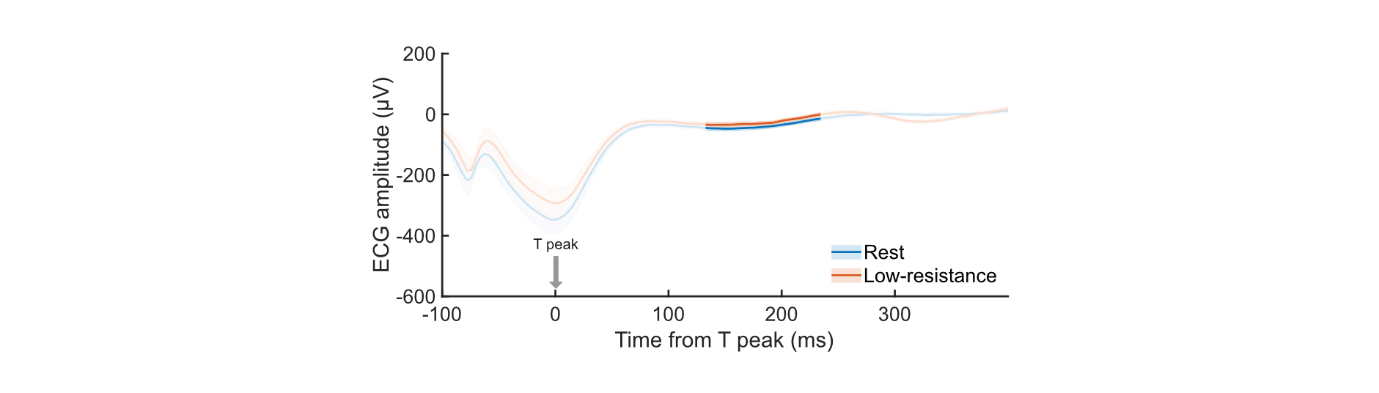  Figure S4 – Control for volume conduction |
| --- |
| T-peak-locked ECG, grand-average across subjects for the rest and low-resistance cycling conditions in the same time window as the HEP analysis. The shaded areas denote the SEM for each time course. There were no significant differences between the two conditions. |

Table S3 – Linear mixed-effects model examining the main effects of exercise intensity condition and heart rate on HEP amplitude

Fixed and random effect results from the LME. The two exercise intensity conditions are rest and low-resistance cycling. The model revealed no significant effect of condition on HEP amplitude after controlling for heart rate (*p* = 0.43), while heart rate showed a marginal negative association with HEP amplitude (*p* = 0.17). Random intercepts for subjects accounted for individual variability in HEP amplitude.

HEP amplitude ~ 1 + Exercise Intensity condition + Heart Rate + (1|Subject)

**Model fit statistics**

| **AIC** | **BIC** | **LogLikelihood** | **Deviance** |
| --- | --- | --- | --- |
| 164.87 | 175.17 | -77.44 | 154.87 |

**Fixed Effects**

| **Predictor** | **Estimate (*b*)** | **SE** | ***t*-value** | ***p*-value** | **95% CI (lower, upper)** |
| --- | --- | --- | --- | --- | --- |
| Intercept | 1.85 | 0.99 | 1.87 | 0.07 | (-0.13, 3.83) |
| Condition (Rest vs. Low-resistance cycling) | 0.20 | 0.25 | 0.80 | 0.43 | (-0.31, 0.71) |
| Heart rate | -0.03 | 0.01 | -2.45 | 0.17 | (-0.05, 0.01) |

**Random Effects**

| **Group** | **Random Effect** | **Estimate** | **95% CI**  **(lower, upper)** |
| --- | --- | --- | --- |
| Subject (Intercept) | Standard deviation | 0.39 | (0.14, 1.12) |
| Residuals | Standard deviation | 0.84 | (0.65, 1.09) |

| 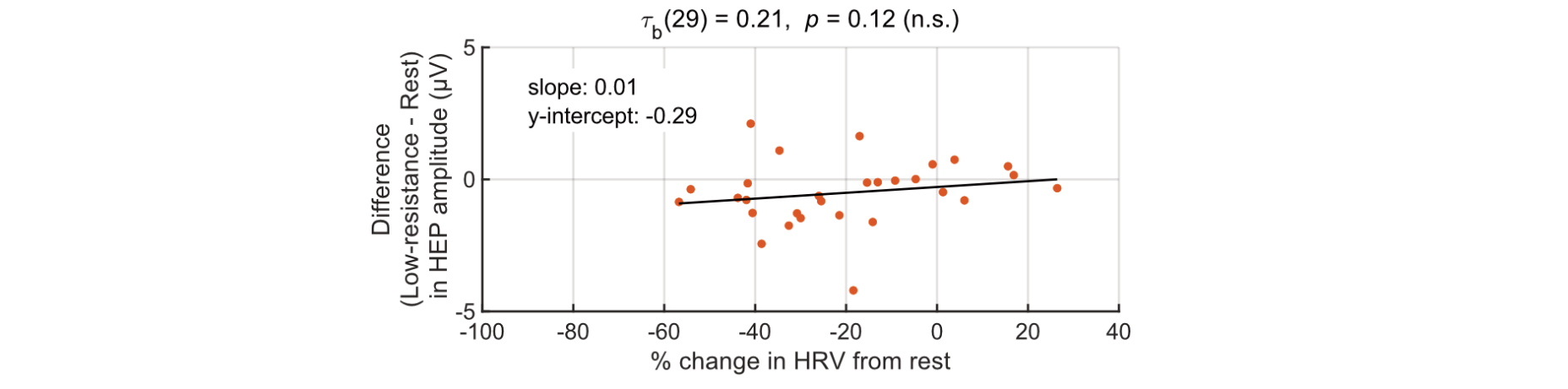  Figure S5 – No statistically significant correlation between cycling-related changes in RMSSD heart rate variability (HRV) and the change in HEP amplitude |
| --- |
| Correlation as assessed by Kendall’s Tau-b between exercise-induced change in HRV and change in HEP amplitude for low-resistance cycling from rest. *N* = 29. |

| 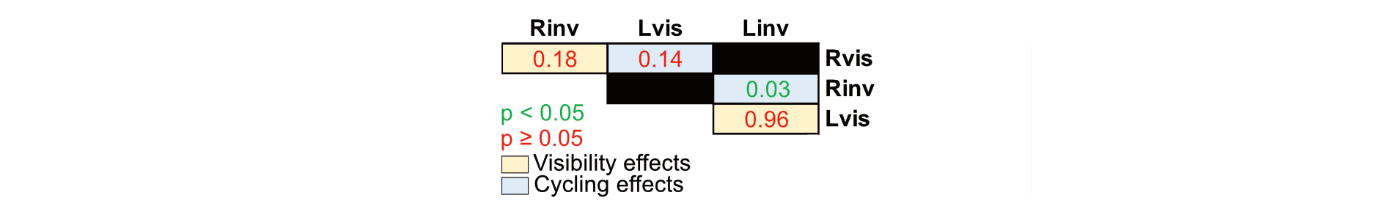  Table S4 - Table of uncorrected *p* values for pairwise comparison of HEP amplitudes across exercise intensity and visibility conditions |
| --- |
| These uncorrected *p* values correspond to the comparisons reported in **Figure 9**, testing for the effects of target visibility (levels: visible – vis; invisible - inv) and exercise intensity (levels: rest – R; low-resistance – L) on HEP amplitudes with Wilcoxon signed-rank tests. There is a significant difference in HEP amplitudes between the rest and low-resistance conditions in trials where the target is rendered invisible. |
